# Suppression subtractive hybridization profiles of radial growth phase and metastatic melanoma cell lines reveal novel potential targets

**DOI:** 10.1186/1471-2407-8-19

**Published:** 2008-01-22

**Authors:** Josane F Sousa, Enilza M Espreafico

**Affiliations:** 1 Department of Cellular and Molecular Biology and Pathogenic Bioagents of the Faculty of Medicine of Ribeirão Preto – University of São Paulo, Ribeirão Preto, SP, Brazil

## Abstract

**Background:**

Melanoma progression occurs through three major stages: radial growth phase (RGP), confined to the epidermis; vertical growth phase (VGP), when the tumor has invaded into the dermis; and metastasis. In this work, we used suppression subtractive hybridization (SSH) to investigate the molecular signature of melanoma progression, by comparing a group of metastatic cell lines with an RGP-like cell line showing characteristics of early neoplastic lesions including expression of the metastasis suppressor *KISS1*, lack of αvβ3-integrin and low levels of *RHOC*.

**Methods:**

Two subtracted cDNA collections were obtained, one (RGP library) by subtracting the RGP cell line (WM1552C) cDNA from a cDNA pool from four metastatic cell lines (WM9, WM852, 1205Lu and WM1617), and the other (Met library) by the reverse subtraction. Clones were sequenced and annotated, and expression validation was done by Northern blot and RT-PCR. Gene Ontology annotation and searches in large-scale melanoma expression studies were done for the genes identified.

**Results:**

We identified 367 clones from the RGP library and 386 from the Met library, of which 351 and 368, respectively, match human mRNA sequences, representing 288 and 217 annotated genes. We confirmed the differential expression of all genes selected for validation. In the Met library, we found an enrichment of genes in the growth factors/receptor, adhesion and motility categories whereas in the RGP library, enriched categories were nucleotide biosynthesis, DNA packing/repair, and macromolecular/vesicular trafficking. Interestingly, 19% of the genes from the RGP library map to chromosome 1 against 4% of the ones from Met library.

**Conclusion:**

This study identifies two populations of genes differentially expressed between melanoma cell lines from two tumor stages and suggests that these sets of genes represent profiles of less aggressive versus metastatic melanomas. A search for expression profiles of melanoma in available expression study databases allowed us to point to a great potential of involvement in tumor progression for several of the genes identified here. A few sequences obtained here may also contribute to extend annotated mRNAs or to the identification of novel transcripts.

## Background

Melanoma arises from melanocytes, specialized cells in the skin responsible for synthesizing and distributing the pigment melanin. This tumor is one of the most aggressive malignancies, marked by elevated capacity to metastasize and by high drug resistance [for review see [1-3]]. Melanoma often arises from inherited or simple acquired nevi, which are pigmentary melanocytic lesions that can progress through hyperplastic to dysplastic nevi and culminate in some cases in the radial growth phase melanoma (RGP), an early melanoma lesion that is confined to the epidermis [for review see [4]]. This lesion usually further progress to a vertical growth phase (VGP) melanoma, in which the cells that were growing only laterally in the epidermis become able to invade into the dermis and acquire metastatic potential [for review see [4]]. The establishment of metastasis is believed to require few additional genetic changes, once cells presenting metastatic phenotype can be readily selected from most VGP melanomas [5].

Cell lines derived from RGP, VGP and metastatic melanoma represent an interesting experimental model for identification and characterization of genes involved in melanoma development, since they sustain *in vitro *the characteristics representing the original state of the tumor stage from which they are derived [6,7]. RGP cell lines usually mimic early, less aggressive melanoma lesions, since they show low anchorage-independent growth, high growth factor dependency, and are non-tumorigenic or have limited ability to induce tumor in immunodefficient mice [6,8]. VGP lesions usually contain cells that have already acquired metastatic capacity and so they show behavior and expression profiles similar to cells from metastasis [4,9]. However, since the tumor lesions are heterogeneous, some cells derived from VGP tumors can still sustain a less aggressive phenotype [5,10]. The heterogeneity of VGP cells was strengthened by the result of a microarray study using melanoma cell lines, in which some VGP cells clustered with metastatic melanoma whereas others did so with RGP cells [11].

In spite of the fact that different lines of evidence support the notion that cancer progresses through discrete phenotypic stages marked by a stepwise acquisition of oncogenic alterations, recent evidence from high-throughput gene expression studies in cancer [12] lead to an emerging paradigm that tumor aggressiveness is intrinsically associated to the mechanisms of tumor birth [13]. From this point of view, higher proclivity towards a metastatic phenotype would be inherent to the initial set of genetic alterations that generate a tumor. The tendency of melanoma to generate metastasis may as well be corroborated by the fact that this tumor derives from melanocytes, naturally migratory neural crest descendants, as suggested by Gupta et al. [14]. The notion that a metastatic melanoma may arise from an RGP lesion has been also supported by clinical and molecular evidence [15,16], strengthening the importance of determining molecular alterations that distinguish particularly less aggressive melanoma cells from metastatic cells as an approach to identify molecular events that drive the selection towards one of these phenotypes.

Many genes with altered expression associated to melanoma progression have been identified [3,4]. A notable example of a molecular marker of the transition from RGP to VGP melanomas is the β3 subunit of the αvβ3 integrin, a vitronectin receptor. The expression of β3 integrin is detected in most VGP and metastatic melanomas, whereas normal melanocytes and RGP melanomas do not express this integrin subunit [17]. The expression of β3 integrin has been shown to contribute to metastatic phenotype by altering the adhesion and promoting survival of melanoma cells [8,18,19]. Another example of a gene involved in the transition from RGP to VGP is *KISS1*, which has been postulated as a metastasis suppressor gene [20,21]. *KISS1 *encodes the protein kisspeptin-1/metastin that was identified as the endogenous ligand for the G protein-coupled receptor GPR54/KISS1R [22,23], and has been shown to play an anti-migratory role *in vitro *and to act as a metastasis inhibitor *in vivo *[20]. *KISS1 *expression is detected in normal melanocyte and RGP melanomas, but its expression is lost in VGP and metastatic cells [20,21]. Also, in other cancer types such as breast, bladder and pancreatic cancer, loss or reduced expression of *KISS1 *has been associated to the metastatic phenotype [24-26]. On the other hand, *RHOC *has been identified as an overexpressed gene in metastatic murine and human melanoma cells in comparison with the non-metastatic parental cells [27]. RHOC, like the other RHO family proteins, is involved in the regulation of the actin cytoskeleton dynamics and overexpression of RHOC induces the cells to become highly metastatic by enhancing their migratory and invasive capacities [27].

Although many cancer-related genes have been characterized, several lines of evidence suggest that many more remain to be identified. Present estimate has indicated that around 1% of the genes in the human genome are involved in cancer and there are predictions that 5–10% or more can contribute to oncogenesis [28]. Suppression subtractive hybridization is a widely used method for separating mRNA sequences that distinguish two mRNA populations [29]. A key feature of the method is the simultaneous normalization and subtraction steps. The normalization step equalizes the abundance of mRNA within the target population, and the subtraction step excludes sequences that are common to the two populations being compared. The SSH methodology allows the detection of low copy transcripts and, in contrast to microarray analysis, it allows the identification of unknown genes or non-coding RNAs, thus representing an alternative and complementary approach for differential expression analyses (for a comparative study see [30]).

In the present work, we used SSH approach to compare a non-tumorigenic cell line whose behavior and expression profile of a particular set of genes are suggestive of low aggressiveness to a pool of established, highly tumorigenic and metastatic cell lines, aiming to identify sets of genes potentially involved in maintaining low versus high aggressiveness status in melanoma.

## Methods

### Cell culture

The cell lines used in this work represent the three major stages of melanoma progression: radial growth phase (WM35, WM1552C and WM1789), vertical growth phase (WM278, WM793 and WM902) and metastasis (WM9, WM852, 1205Lu and WM1617). All melanoma cell lines were kindly provided by Dr. Meenhard Herlyn (Wistar Institute, Philadelphia, PA). The cells were maintained in melanoma medium, consisting of four parts of MCDB153 (Sigma, Saint Louis, MO, USA) and one part of L-15 (Invitrogen, Carlsbard, CA, USA), supplemented with 2 mM CaCl_2_, 5 μg/ml insulin and 2% fetal bovine serum (Invitrogen, Carlsbard, CA, USA).

### Isolation of RNA and mRNA

Total RNA was isolated using Trizol reagent (Invitrogen, Carlsbard, CA, USA) and mRNA was isolated from total RNA using the Oligotex™ mRNA kit (Qiagen, Valencia, CA, USA) according to manufacturer's instructions. The integrity of RNA and mRNA was checked on a 1% formaldehyde agarose gel.

### Suppression subtractive hybridization (SSH)

The subtractive libraries were constructed using the Clontech PCR-Select™ cDNA Subtraction kit (Clontech, Palo Alto, CA, USA). Briefly, 1 μg of mRNA (poly dA^+ ^RNA) from WM1552C (RGP-like) and equal amount from a pool of four metastatic cell lines (WM9, WM852, 1205Lu and WM1617) were used for double strand cDNA synthesis, and the resulting cDNA was digested with *Rsa *I. For the RGP-library, the digested cDNA from WM1552C (as a Tester) was split into two groups and linked to either adaptor I or adaptor 2R. Subtractive hybridization was performed by annealing an excess of the metastatic cell cDNA (as a Driver) with each sample of adaptor-ligated tester cDNA. The cDNAs were heat denatured and incubated at 68°C for 8 hours (h). After the first hybridization, the two samples were mixed together and hybridized again with freshly denatured driver cDNA for 20 h at 68°C. The two rounds of hybridization would generate a normalized population of tester specific cDNAs with different adaptors on each end. After filling in the ends, two rounds of PCR amplification were performed to enrich for the desired cDNAs containing both adaptors. The optimized cycling for the first and second PCR rounds, to increase representation and reduce redundancy of subtracted cDNAs, were 27 and 10 cycles, respectively. The Met-library was constructed using the same approach but with cDNA from the metastatic cell lines as a Tester and cDNA from WM1552C as a Driver.

### Cloning of the subtracted cDNAs

The amplified products containing the subtracted cDNAs from both subtraction processes (4 μL) were independently ligated into a pGEM-Teasy vector (Promega Co., USA) and transformed into *E. coli *strain DH5α. Bacteria were supplied with 800 μL of SOB medium, incubated for 1 h at 37°C, and subsequently plated onto agar plates containing 100 μg/mL ampicillin, 100 mM IPTG and 100 mg/mL X-gal at 37°C, for 20 h. White colonies were inoculated into 96-well plates containing 150 μL of 2× YT liquid medium supplemented with 100 μg/mL ampicillin. The cultures were grown overnight, without shaking, at 37°C. PCR amplification to check for the positive clones, i.e., to confirm the presence of insert, and to generate sequencing templates was performed as previously described [31].

### Sequencing, annotation and sequence analysis

A total of 753 clones from both libraries were sequenced using the kit DYEnamic ET dye terminator cycle sequencing (Amersham-Pharmacia, Pollards Wood, UK) and a M13 primer in the capillary DNA sequencer Megabace 1000 (Amersham-Pharmacia Biotech, Pollards Wood, UK). The BLAST program was used to search for the cDNA sequence similarity of isolated clones in the GenBank [32]. Annotated sequences were submitted to functional annotation according to the Gene Ontology database, using the tool GOTM-Gene Ontology Tree Machine [33]. For the chromosome distribution analysis, chromosome locations of all genes/ESTs were obtained from GenBank accession number reports or through BLAT alignment [34], and then the total number of genes per human chromosome for each library was plotted in a bar graphic. Graphics showing the gene distribution along each human chromosome was generated using the "Chromosomal Distribution Chart" tool from the WebGestalt home page [35].

### Northern blot

For Northern blot preparation, 20 μg of total RNA was separated by 1% formaldehyde-agarose gel electrophoresis and transferred to nylon membrane (Hybond N, Amersham Pharmacia Biotech, Pollards Wood, UK) by standard methods. RNA was fixed to membrane by baking the blot and by UV cross-linking. Pre-hybridization was done in a solution containing 7% SDS, 1% BSA, 1 mM EDTA, and 0.5 M NaHPO_4 _pH 7.5 [36], at 65°C for at least 1 h, in a 30/3,5 cm roller bottle in a hybridization oven. For probe generation, digested inserts were gel purified (Qiaex II kit-Qiagen, Valencia, CA) and about 50 ng were radio-labeled with [α-32P]-dCTP by random-priming (Rad-prime kit, Invitrogen, Carlsbad, CA, USA). Unincorporated nucleotides were removed by gel filtration through a G-50 Sephadex column. The hybridization was performed for 18 h using the probe to 1 × 10^6 ^CPM/ml hybridization solution. The blots were washed in the following manner: one time in 2 × SSC, 0,2% SDS, for 5 min, at room temperature; two times in 1 × SSC, 0,2% SDS, for 30 min each, at 65°C; and one time in 0,2 × SSC, 0,2% SDS, for 30 min, at 65°C. Then, the blots were covered in clear plastic wrap and exposed to a Phosphoimager screen (Molecular Dynamics, Piscataway, NJ, USA). In order to correct for different lane loads, blots were stripped at 100°C in 0.5% SDS and probed with a fragment for *ACTB *(β actin) gene.

### RT-PCR for *HLA-DRA *gene

For RT-PCR, total RNA was treated with DNase I (Promega, Madison, WI, USA) at 1 U/2 μg of total RNA in 10 μL reaction volume and incubated for 30 min at 37°C, followed by enzyme inactivation by addition of 1 μL of 20 mM EDTA and incubation for 15 min, at 65°C. cDNA synthesis was performed using 2 μg of total RNA in 20 μL reaction with Superscript II Reverse Transcriptase (Invitrogen, Carlsbad, CA, USA), according to the manufacture's instructions, using 4 μL of 5× first-strand buffer, 1 μL of 10 mM dNTP, 200 U Superscript II enzyme, 2 μL of 0.1 M DTT, and 250 ng oligo dT primer (Invitrogen, Carlsbad, CA, USA). For PCR reactions, 1 μL of each synthesized cDNA was used as template in a reaction volume of 50 μL containing 200 μM dNTPs, 1,5 mM MgCl_2_, 0.25 μM each primer, and 1 U Taq DNA polymerase in the manufacture's recommended buffer (Invitrogen, Carlsbad, CA, USA). The reaction was allowed to denature for 4 min at 94°C, followed by amplification (25, 28, 30 e 32 cycles: 45 s at 94°C, 1 min at 55°C, 1 min at 72°C). At indicated cycles, a 5 μL sample was colleted from each reaction. Amplification of *ACTB *(β actin) cDNA was done as control for mRNA content. The following forward (F) and reverse (R) primers were used: F-ACAGAGCGCCCAAGAAGAAAA and R-CTCAAAGCTGGCAAATCGTC for amplification of *HLA-DRA*; and F-GGCATCGTGATGGACTCCG and R-GGAAGGTGGACAGCGA for *ACTB*. PCR products were loaded onto a 1% agarose gel and electrophoresed in TAE buffer. Gels were subjected to ethidium bromide staining and were imaged in a UV transilluminator using a digital Kodak camera.

### Analysis of the expression profile of genes represented by subtractive clones in a publicly available microarray study of melanoma samples

We downloaded from the PNAS website [37] the table number 10 containing the normalized and log_2 _transformed expression data from the microarray study described by Haqq et al [16]. This table presents the data of the comparison of expression profiles of samples from normal skin, nevi, primary and metastatic melanomas using a microarray from Research Genetics containing 20,862 human cDNA clones. Using a locally developed computer script, we extracted the expression data from their microarray analysis for all genes that were also represented in both of our subtractive libraries (RGP and Met). The expression values of both groups of genes were submitted to SAM (Significance Analysis of Microarrays) [38] software in a two class comparison, first to detect the genes presenting differential expression between primary and metastatic melanomas and then, in a extended analysis, between non-neoplastic tissues (skin and nevi) and tumors (primary and metastatic melanomas). The results of SAM were extracted using the software SAMSTER [39], submitted to hierarchical clustering using CLUSTER and then visualized by JAVATREEVIEW [40].

## Results

### Selection of cell lines for generation of two cDNA SSH libraries and sequencing analysis

We used the SSH approach to identify populations of mRNA that distinguish between a non-tumorigenic RGP cell line (WM1552C) and a pool of four metastatic cell lines (WM9, WM852, WM1617, 1205Lu). In order to reduce individual genetic variations, we initially aimed to use a pool of less aggressive cell lines as we do for the metastatic cells. However, as shown here based on several criteria we failed to find more than one among six RGP/VGP cell lines tested to fit in a "less aggressive" phenotype. Also, we were discouraged to include a VGP cell line in the study based on the rationale that cells from VGP tumors are more heterogeneous, as pointed out in the Background section.

**Figure 1:**
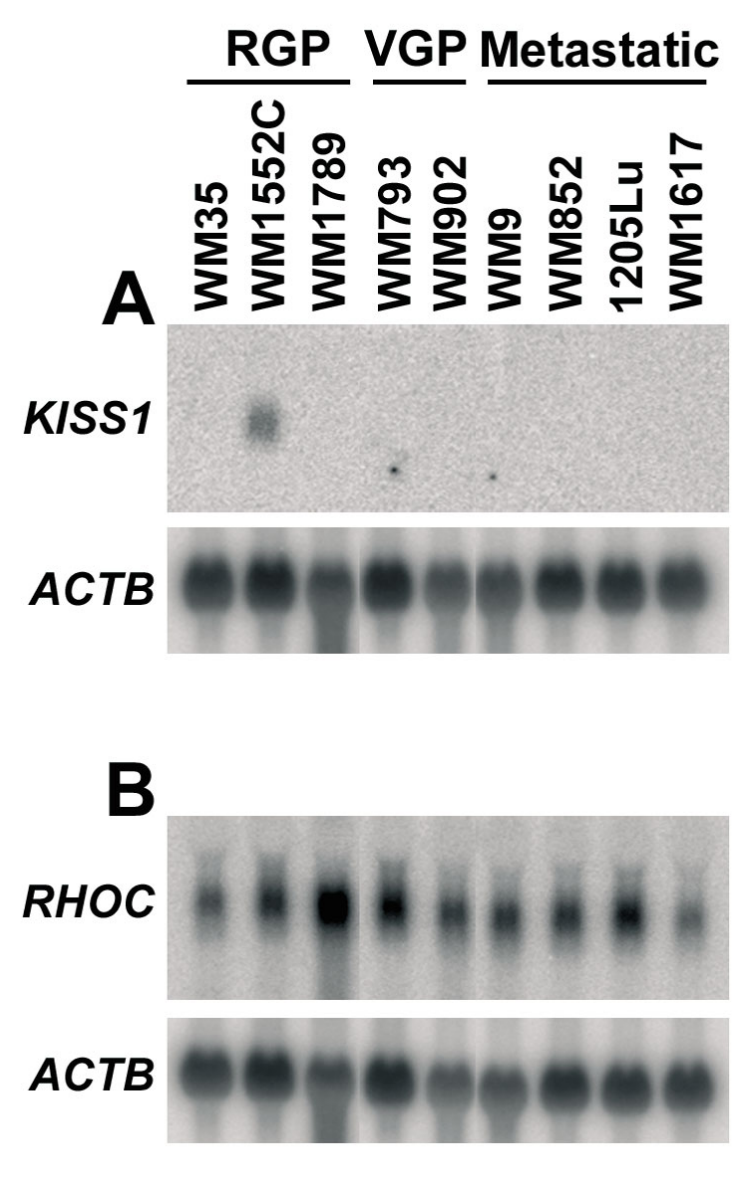
***KISS1 *and *RHOC *mRNA expression in a panel of RGP, VGP and metastatic melanoma cell lines**. Comparison of the expression levels of the *KISS1 *metastasis suppressor gene (**A**) and the small GTPase *RHOC *(**B**) among melanoma cell lines of different stages of tumor progression supported the selection of WM1552C cell line as the RGP representative for suppression subtractive hybridization against a pool of metastatic cell lines. Samples of 20 μg of total RNA from different melanoma cell lines were submitted to electrophoresis in 1% agarose-formaldehyde gel and transferred to nylon membrane (Hybond N, Amersham Pharmacia Biotech) by standard methods. Fragments of the indicated genes were radiolabeled with [α-32P]-dCTP by random-priming (Rad-prime kit, Invitrogen) and used as probes for Northern blot hybridization. In order to correct for loading differences, after stripping, the blots were probed with a ACTB (β-actin) cDNA fragment.

For selecting the cell lines, we checked on the expression of three known molecular markers of melanoma progression, *KISS1 and RHOC *mRNAs and the αvβ3 integrin, in the panel of melanoma cell lines used here. *KISS1 *mRNA expression was detected by Northern blot only in the RGP cell line WM1552C (Fig. 1A). So even the other two RGP cell lines (WM35 and WM1789) included in our study failed to show detectable levels of *KISS1 *mRNA, although by using a more sensitive method (RT-PCR/Southern blotting), a weak expression of *KISS1 *transcript in the WM35 cell line in contrast with lack of expression in WM793 (VGP) and 1205Lu (metastatic) was previously reported [21].

The *RHOC *mRNA was detected in all melanoma cell lines, presenting an elevated expression particularly in the RGP cell line WM1789 (Fig. 1B). Also, WM1552C was previously shown to lack expression of αvβ3 integrin [8] in contrast to WM793 and the metastatic cell lines WM9 and 1205Lu [41]. Here, we confirmed by flow cytometry that WM1552C cells do not express αvβ3 integrin, while we observed expression of this integrin in WM35 (RGP), WM278 (VGP) and WM1617 metastatic cells (data not shown). In addition, we found that expression of these molecular markers was compatible with observations made in our laboratory (unpublished data) that WM1552C is more sensitive to apoptosis triggered by cell adhesion impairment (anoikis) than WM35 cells and that both WM35 and WM1789 cell lines were capable to generate slowly growing tumor in SCID mice, in contrast with WM1552C that in a preliminary assay was unable to induce visible primary tumor when injected (2 × 10^6 ^cells) into SCID mice in the same conditions. Furthermore, spontaneous transformation towards a more malignant phenotype has been pointed out for WM35 cell line [7] and, indeed, in contrast to WM1552C, WM35 cells were recently shown to express the melanoma chondroitin sulfate proteoglycan (MCSP), a surface molecule implicated in enhanced tumor migration, invasion and anchorage-independent survival [42]. In view of these contrasts, although WM1552C cells carry the *BRAF *mutation V599E, they appears to retain the phenotype of a less aggressive melanoma tumor as compared with the other cell lines of this collection and therefore it was the only cell line selected as representative of the RGP stage for this study.

**Figure 2:**
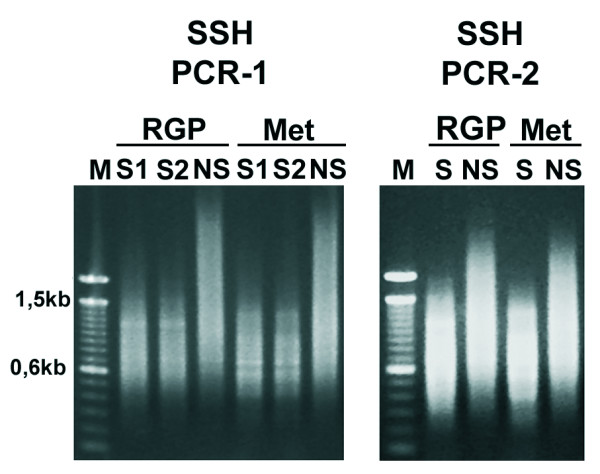
**Subtracted cDNA profiles of the RGP and metastatic (Met) cells**. PCR-1 represents the PCR products generated using a single primer directed towards both adaptors, after 27 amplification cycles from two duplicate samples of subtracted (S1 and S2) or non-subtracted (NS) cDNA of the RGP (WM1552C) and the metastatic (a pool of WM9, WM852, 1205Lu and WM1617) cell lines. PCR-2 represents the PCR product generated after 10 amplification cycles by nested-PCR using a specific primer for each adaptor. Note the difference between the subtracted and non-subtracted profiles.

Two subtracted cDNA collections were obtained, one of cDNA from the RGP cell line WM1552C subtracted from a cDNA pool of four metastatic cell lines (WM9, WM852, 1205Lu and WM1617), which we named RGP library. The second library, referred as Met library, was obtained by the reverse subtraction. The cDNA profiles generated by the subtraction process are shown in Fig. 2. Cloning of these cDNAs into the pGEM vector allowed us to obtain 2016 clones for the RGP library and 1920 clones for the Met library. PCR analysis of 395 and 336 randomly selected clones from the RGP and Met libraries, respectively, indicated that 97% of the clones from the RGP library and 98% from the Met library contained inserts and that the insert size of most clones was ≥ 600 bp (images of representative agarose gels for each library are shown in the Additional File 1, Fig. S1).

A total of 753 clones from both libraries were sequenced and annotated, as summarized in Table 1. The sequences were submitted to GenBank [GenBank accession numbers: ES315683–ES316435]. Most sequences (94–95%) corresponded to annotated mRNA sequences. The sequence redundancy within each library is low, 288 different genes are represented in the RGP library (non-redundancy of 82%) and 217 in the Met library (non-redundancy of 59%). Additional File 2 lists the identifier and annotation of the genes represented in the RGP (Tables S1) and Met (Table S2) libraries and indicates the number of sequence occurrences for each gene in the library. Common to both libraries, there were 10 genes represented by 22 sequences, which corresponded to only 2.5% of the total number of sequences analyzed (Table 1, and Additional File 2). A total number of 24 and 19 sequences from the RGP and Met libraries, respectively, match only EST (Expressed Sequence Tag) sequences in the GenBank, thus suggesting that the libraries may contain sequences representing rare human transcripts. In addition, 8 RGP sequences and 5 Met sequences mapping to the human genome sequence (Additional File 3, Fig. S2–S14) do not match any expressed sequences and thus they might represent novel transcripts. Even among the sequences matching known human mRNAs we obtain additional information. For example, a sequence from the Met library aligns to and extends a putative alternative exon of a cDNA corresponding to the gene *ABCB5 *(Additional File 3, Fig. S15).

**Table 1:** Global analysis of the clones generated by Suppression Subtractive Hybridization

**SSH collections**	**RGP library**	**Met library**
Number of clones obtained	2016	1920
Sequences analyzed	367 (18.5%)	386 (20.5%)
Sequences matching human mRNAs/ESTs	351 (94.1%)	368 (95%)
Sequences matching introns	5 (1.3%)	1 (0.26%)
Chimerical clones	2 (0.5%)	2 (0.5%)
Sequences matching intergenic regions	4 (1.1%)	4 (1.0%)
Sequences matching mitochondrial genome	5 (1.3%)	11 (2.8%)
Number of genes represented	288 (82%)	217 (59%)
Genes represented by more than one clone	37 (11%)	32 (8.7%)
Sequences corresponding to genes common in both libraries	12 (2.2%)	10 (2.5%)

Assuming that the genes represented by multiple clones within each library are the ones with the highest differential expression levels between the RGP and metastatic cell lines, we reviewed the literature on these genes by searching for their involvement in cancer in general and specifically in melanoma, as summarized in the Additional File 4 (Tables S3 and S4). In the RGP library, 37 genes are represented by at least 2 sequences (maximum number of clones for a gene is 10) and in the Met library this number is 32 genes (maximum number of clones for a gene is 55). Among these 37 genes from the RGP library, 18 have been reported with some alteration in cancer (only 3 of them in melanoma) whereas the 19 remaining have not been associated to cancer. In the Met library, 23 from the 32 genes have been associated to cancer, including 10 also associated to melanoma development.

### Validation of the expression pattern of genes identified in the subtractive libraries

**Figure 3:**
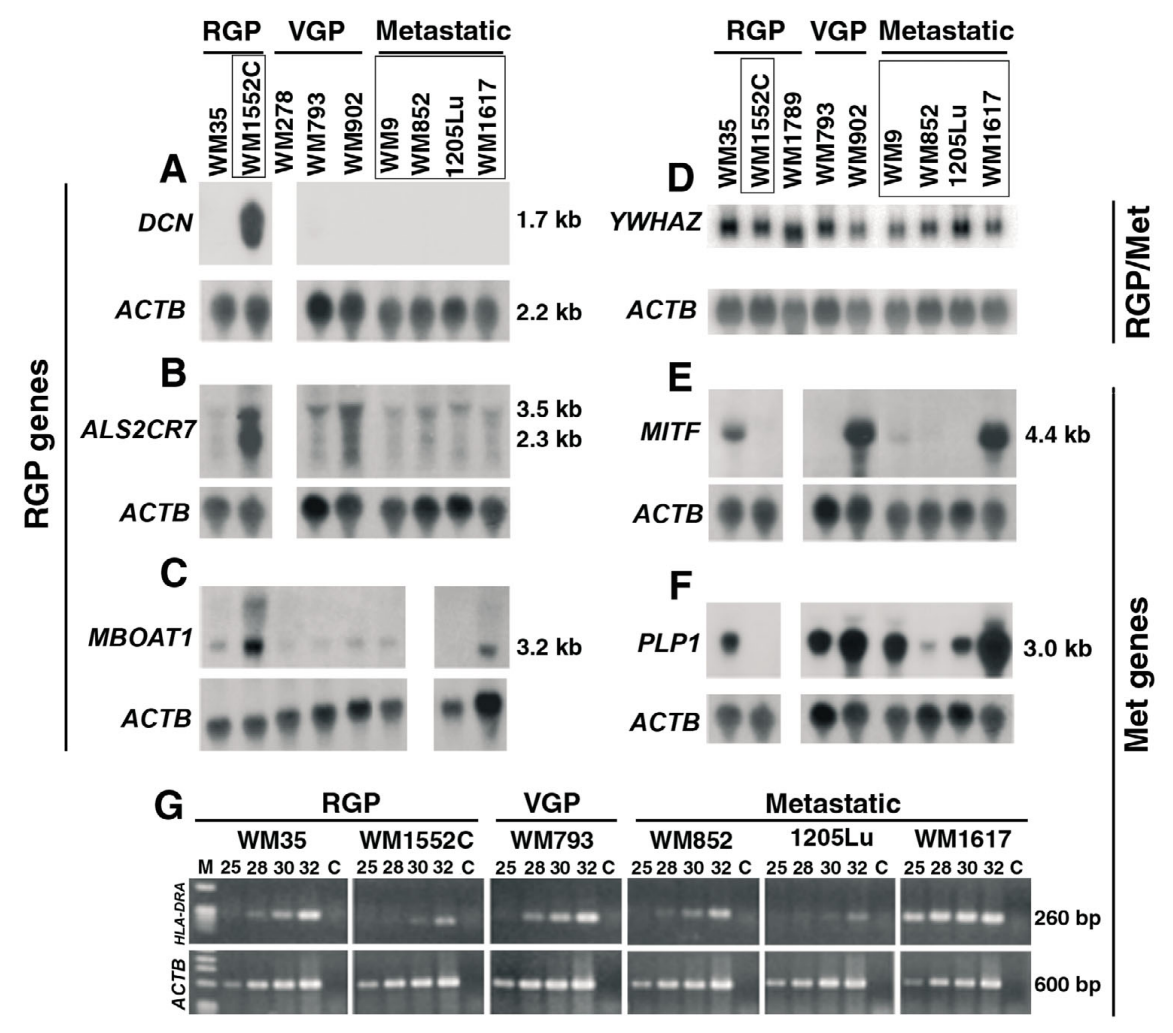
**Validation by Northern blot and RT-PCR of the expression pattern of seven genes identified in the SSH libraries**. Frames depict the names of cell lines used in the construction of the libraries. The inserts of cDNA clones corresponding to the genes *DCN *(decorin) (**A**), *ALS2CR7 *(**B**) and *MBOAT1 *(**C**) of the RGP library; *YWHAZ *(14-3-3 ξ) (**D**) identified in both libraries; and *MITF *(**E**) and *PLP1 *(**F**) from the Met library were isolated and used as probes for hybridization in Northern blots containing total RNA from the melanoma cell lines indicated above the panels – **Blank lanes **mean that the corresponding cell line was not included in the Northern blot, and were introduced to allow alignment among panels. Northern blots were prepared as described in Figure 1. *HLA-DRA *(**G**) identified in the Met library was validated by RT-PCR. For RT-PCR, total RNA samples (2 μg) from the indicated cell lines were, after DNase treatment, submitted to reverse transcription with Superscript II (Invitrogen) using oligo dT as primer and the cDNA was used as template for PCR amplification with *HLA-DRA *primers. After 25, 28, 30 and 32 amplification cycles, 5 μl aliquots were collected for agarose gel electrophoresis. As endogenous control, a pair of primers for the *ACTB *(β-actin) mRNA was used. **C**: Control RT-PCR amplification using as template RNA (DNase treated) without prior reverse transcription.

The identification of only 2% of the clones shared by both libraries strongly suggested that the cDNA subtraction was highly efficient. However, to confirm that this was indeed the case, we selected 7 genes for validation by Northern blots and RT-PCR in a panel of 6–8 melanoma cell lines that represent the three stages of tumor progression, including the cell lines used for the SSH libraries (Fig. 3). The genes selected for validation from the RGP library were *DCN *(represented by 8 clones), *ALS2CR7 *(10 clones) and *MBOAT1 *(3 clones). *DCN *encodes decorin, a secreted protein involved in cell growth regulation and apoptosis induction in tumors [43]. By Northern blot (Fig. 3A), we detected *DCN *mRNA only in the WM1552C cells and at very high levels, indicating that these libraries represent genes highly differentially expressed. *ALS2CR7*, a candidate gene of amyotrophic lateral sclerosis 2, encodes a putative protein kinase, based on Gene Ontology prediction, with no characterized function. As shown in Fig. 3B, although some signal for *ALS2CR7 *mRNA expression was detected in all eight cell lines analyzed, all four metastatic cell lines presented equivalently low signals and the highest levels were detected in WM1552C, confirming the differential expression of this gene identified in the RGP SSH library. The *MBOAT1 *gene encodes a hypothetical transmembrane protein containing an O-acyltransferase domain, also with no characterized function and as predicted by its occurrence in the RGP library, we confirmed that its mRNA expression is higher in WM1552C (Fig. 3C). On the other hand, when a fragment of a gene identified in both libraries, *YWHAZ *(14.3.3ζ) was used as probe for Northern blot hybridization (Fig. 3D), we detected average signals of similar intensity between the RGP WM1552C and the metastatic cell lines, although some variation in the expression of this gene can be noted among the cell lines analyzed.

From the Met library, we selected the genes *MITF *(represented by one clone), *PLP1 *(24 clones) and *HLA-DRA *(55 clones). MITF is a transcription factor involved in melanocyte differentiation and survival and even though it is represented by only one sequence in the Met library, its differential expression between the RGP WM1552C and the metastatic cell lines was confirmed by Northern blot (Fig. 3E). High levels of *MITF *mRNA were detected in three of the cell lines independent of the growth phase, the RGP WM35, the VGP WM902, and the metastatic WM1617, but no signal was detected in the RGP WM1552C cells. *PLP1 *encodes a proteolipid protein involved in myelinization and as predicted by its presence in the Met library, the RGP WM1552C cell line showed no detectable expression of *PLP1 *mRNA while high levels were detected in the metastatic cell lines (Fig. 3F). To validate the expression pattern of the MHC class II *HLA-DRA*, the most redundant gene found in the Met library, we performed RT-PCR using cDNA from 6 cell lines and analyzed the amplified product at 25, 28, 30 and 32 amplification cycles on agarose gel (Fig. 3G). The data confirmed high expression levels of *HLA-DRA *mRNA in the WM1617 metastatic cell line and a weak expression in the RGP cell line WM1552C (Fig. 3G). Moderate expression levels were detected in cell lines of different growth phases (WM35, WM793 and WM852) and, interestingly, 1205Lu, which was selected in immunodefficient mouse from WM793, shows low expression levels. Also, high levels of the HLA-DRA protein were confirmed by flow cytometry for WM1617 and WM9 (data not shown). Therefore, the melanoma cell lines analyzed here express differential levels of *HLA-DRA *but without showing correlation to any particular phase of the tumor development. In summary, we conclude that all of the genes selected for validation confirmed the expression pattern predicted by their presence in only one of the two libraries.

### Genes of specific biological processes and from distinct chromosome locations are differentially enriched between the RGP and Met libraries

In order to verify if specific classes of proteins are differentially enriched in the RGP or Met libraries, we submitted the two total lists of genes identified in our SSH libraries to a functional annotation based on the Gene Ontology, according to the biological processes. The annotation was performed using the software GOTM (Gene Ontology Tree Machine) that also compare the frequency of genes in each functional class with the expected frequency based on the distribution of all human genes throughout the GO functional classes. The GO functional classes that are significantly enriched in the RGP and Met libraries, in comparison to the distribution of all predicted human genes, are listed in Tables 2 and 3. Genes corresponding to proteins involved in nucleic acid binding are enriched in both libraries, however the number of genes and processes related to this function is greater in the RGP library. In the Met library, regulation of transcription and RNA processing are the two processes involving nucleic acid binding proteins that were considered enriched. In the RGP library, we detected a large number of genes related to DNA metabolism, DNA repair, chromatin remodeling and RNA processing. In addition, proteins involved in cytoskeleton processes related to subcellular transport and localization, as well as proteins involved in macromolecule degradation are also enriched in the RGP library. On the other hand, processes related to cell adhesion and cell migration were considered specifically enriched in the Met library. These processes include genes coding for components of extracellular matrix and several types of receptors such as G protein-coupled receptors, tyrosine kinase receptors, integrins and nuclear receptors.

**Table 2:** Functional classes of genes enriched in the RGP library in comparison to the frequency within the whole set of predicted human genes

**Functional Class (biological process)**	**Genes**	**Relative enrichment significance**
establishment of cellular localization	AP1G1, DYNC1I2, COPZ1, NUP160, KIF5B, RANBP5, PAFAH1B1, FLJ10292, C14orf108, RAN, SET, SGNE1, SSR1, SSR2, BAT1, SEC24C	O = 16^a^; E = 8.04^b^; R = 1.99^c ^P = 0.0067^d^
nucleocytoplasmic transport	NUP160, RANBP5, FLJ10292, RAN, SET, BAT1	O = 6; E = 1.61; R = 3.73; P = 0.0054
organelle organization and biogenesis	ARPC3, DCTN6, MYST2, DDX1, DYNC1I2, XRN2, KIFAP3, DAAM1, POT1, H3F3A, HDAC1, HMGB2, KIF5B, STMN1, PAFAH1B1, ATRX, KLHL4, PXMP3, RAN, SET, SMYD3, WASPIP, ACTL6A, H2AFV,	O = 24; E = 10.92; R = 2.2; P = 0.00022
chromosome organization and biogenesis	MYST2, POT1, H3F3A, HDAC1, HMGB2, ATRX, SET, SMYD3, ACTL6A, H2AFV	O = 10; E = 4.05; R = 2.47; P = 0.0076
microtubule-based process	DYNC1I2, XRN2, KIFAP3, KIF5B, STMN1, PAFAH1B1, RAN	O = 7; E = 2.13; R = 3.29; P = 0.0056
nucleobase biosynthesis	PAICS, PPAT	O = 2; E = 0.12; R = 16.67; P = 0.0064
regulation of protein biosynthesis	DDX1, EIF4B, EIF4G2, PUM2, TLR3, EIF4E2	O = 6; E = 1.66; R = 3.61; P = 0.0064
DNA metabolism	POLD3, MYST2, XRN2, POT1, H3F3A, HDAC1, HMGB2, NONO, ORC2L, ATRX, RAD23B, RAN, SET, SMYD3, UBE2A, XRCC5, HAT1, ACTL6A, H2AFV	O = 19; E = 8.44; R = 2.25; P = 0.00078
DNA packaging	MYST2, H3F3A, HDAC1, HMGB2, SET, SMYD3, HAT1, ACTL6A, H2AFV	O = 9; E = 3.42; R = 2.63; P = 0.0074
DNA repair	POLD3, XRN2, HMGB2, NONO, ATRX, RAD23B, UBE2A, XRCC5	O = 8; E = 2.91; R = 2.75; P = 0.0088
response to DNA damage stimulus	POLD3, XRN2, HMGB2, NONO, ZAK, ATRX, RAD23B, UBE2A, XRCC5	O = 9; E = 3.24; R = 2.78; P = 0.0053
RNA metabolism	SYNCRIP, DDX17, SF3A3, DDX1, DCP2, ELAVL1, XRN2, SF3B1, LSM5, HNRPC, HNRPU, NONO, FLJ10292, RARSL, SNRPG, BAT1, TTF2, SIP1, DDX23	O = 19; E = 5.8; R = 3.28; P = 5.44-06
RNA processing	SYNCRIP, DDX17, SF3A3, DDX1, XRN2, SF3B1, LSM5, HNRPC, HNRPU, NONO, FLJ10292, SNRPG, BAT1, TTF2, SIP1, DDX23	O = 16; E = 4.68; R = 3.42; P = 1.84374398023E-05
RNA splicing	SYNCRIP, SF3A3, DDX1, SF3B1, LSM5, HNRPC, NONO, FLJ10292, SNRPG, BAT1, TTF2, SIP1, DDX23	O = 13; E = 2.01; R = 6.47; P = 1.03E-07
RNA localization	NUP160, FLJ10292, RAN, BAT1	O = 4; E = 0.64; R = 6.25; P = 0.0037
macromolecule catabolism	YME1L1, DDX1, DCP2, ELAVL1, XRN2, USP33, ARIH1, MDH1, PSMA4, PSMA5, PSMB4, PSMB6, UBE2A, USP8	O = 14; E = 4.57; R = 3.06 P = 0.00019

a: observed number of genes in the category; b: expected number of genes in the category; c: observed/expected ratio; d: p-value of the enrichment significance

**Table 3:** Functional classes of genes enriched in the Met library in comparison to the frequency within the whole set of predicted human genes

**Functional Class (biological process)**	**Genes**	**Relative enrichment significance**
cell adhesion	ADAM10, CTGF, CTNNB1, CTNND1, FN1, ITGA6, ITGB1, ITGB8, LAMA4, NRCAM, SPP1, TGFBI, THBS2, TNFAIP6, HMCN1, CD164, NRP2, NRXN3, CD36	O = 19^a^; E = 6.7^b^; R = 2.84^c^; P = 3.71E-05^d^
cell-matrix adhesion	ITGA6, ITGB1, ITGB8, SPP1	O = 4; E = 0.6; R = 6.67; P = 0.0030
regulation of cell adhesion	ADAM10, LAMA4, TGFBI, CD164	O = 4; E = 0.4; R = 10; P = 0.00064
integrin-mediated signaling pathway	ADAM10, ITGA6, ITGB1, ITGB8	O = 4; E = 0.57; R = 7.02; P = 0.0025
intracellular receptor-mediated signaling pathway	CTNNB1, EDD1, RB1, NCOA4	O = 4; E = 0.47; R = 8.51; P = 0.0012
cell differentiation	ACVR1C, DCT, GPM6B, MGP, MITF, NRCAM, SERPINE2, SFRP1, SPP1, TYR, TYRP1, NRP2, NRXN3	O = 13; E = 5.34; R = 2.43; P = 0.0027
cell motility	CTGF, FN1, ITGB1, LAMA4, NRCAM, SERPINE2, SPP1, NRP2, NRXN3	O = 9; E = 2.42; R = 3.72; P = 0.00072
cell migration	FN1, ITGB1, LAMA4, NRCAM, SERPINE2, SPP1, NRP2, NRXN3	O = 8; E = 1.04; R = 7.69; P = 9.41E-06
nucleocytoplasmic transport	ADAM10, KPNA1, NPM1, IPO9, G3BP2, THOC1	O = 6; E = 1.25; R = 4.8; P = 0.0016
negative regulation of cell proliferation	GPNMB, FABP7, IL6, NPM1, CUL5, CD164	O = 6; E = 1.59; R = 3.77; P = 0.0052
aromatic amino acid family metabolism	DCT, TDO2, TYR, TYRP1	O = 4; E = 0.23; R = 17.39; P = 6.642E-05
aromatic compound metabolism	CPM, DCT, TDO2, TYR, TYRP1	O = 5; E = 0.97; R = 5.15; P = 0.0028
cofactor biosynthesis	PBEF1, TMEM131, TPK1, ATP5A1, ATP6V1B2	O = 5; E = 1.18; R = 4.24; P = 0.0066
coenzyme biosynthesis	PBEF1, TMEM131, TPK1, ATP5A1, ATP6V1B2	O = 5; E = 1.04; R = 4.81; P = 0.0039
negative regulation of transcription	HMGB1, TRIM33, HBXAP, NKRF, RB1, ARID5B	O = 6; E = 1.72; R = 3.49; P = 0.0076
positive regulation of transcription	CTNNB1, ILF2, NFATC2, HBXAP, RB1, NCOA4	O = 6; E = 1.19; R = 5.04; P = 0.0012
mRNA processing	DHX8, PABPC1, GRSF1, SFRS2, SNRPB2, SNRPG, G3BP2, THOC1	O = 8; E = 2.13; R = 3.76; P = 0.0013
pigment metabolism	DCT, TYR, TYRP1	O = 3; E = 0.25; R = 12; P = 0.0018

a: observed number of genes in the category; b: expected number of genes in the category; c: observed/expected ratio; d: p-value of the enrichment significance

**Figure 4:**
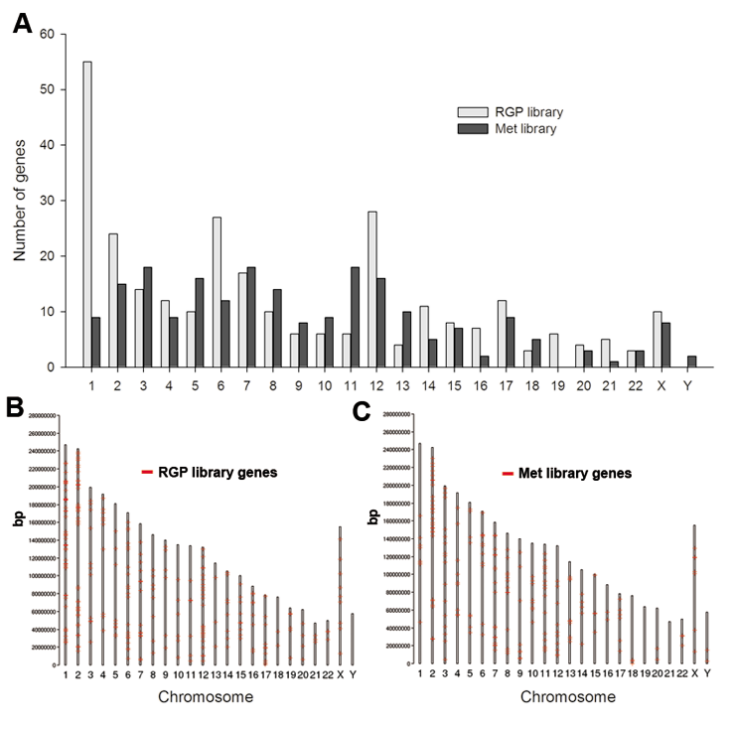
**Genes from distinct chromosome locations are differentially enriched between the RGP and Met libraries**. Chromosome locations of all genes/ESTs were obtained from GenBank accession number reports or through BLAT alignment. (A) Represents the total number of genes per human chromosome for each library; (B and C) Represent the chromosome locations for all genes identified in the RGP (B) and Met (C) libraries, along the length (bp) of all human chromosomes. The absence of genes mapping to Y chromosome in the RGP library is not explained by lack of this chromosome since the RGP cell line WM1552C was obtained from a male patient.

We also analyzed the chromosome location of all genes/ESTs identified in the SSH libraries (Fig. 4). Interestingly, genes mapping to chromosome 1 are much more represented in the RGP library (19%) than in the MET library (4%). Also, at lower extent, chromosomes 2, 6 and 12 had more genes identified in the RGP than in the Met library, whereas genes from chromosomes 11 and 13 showed an inverted pattern.

### Analysis of the expression profile of the genes represented in the SSH libraries in a panel of melanocytic samples using a publicly available microarray study

**Figure 5:**
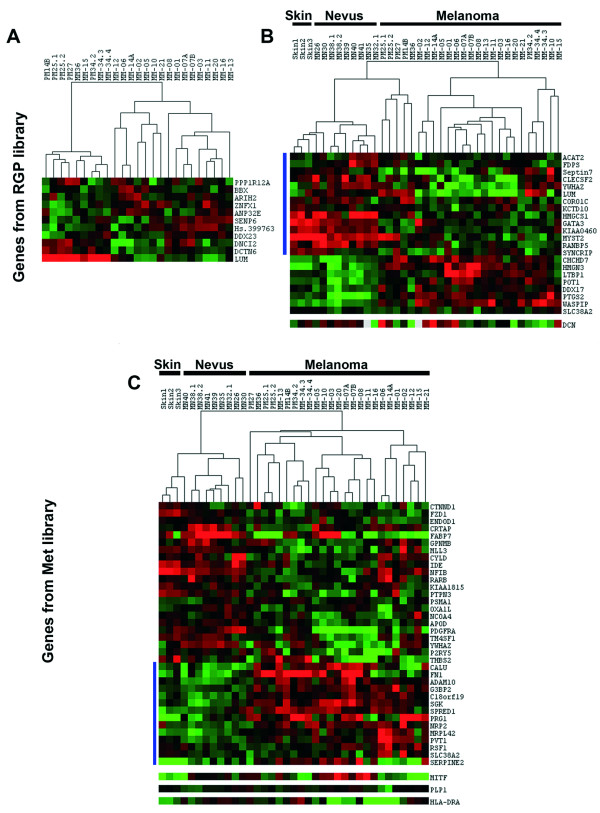
**Genes identified in the SSH library distinguish non-neoplastic from neoplastic tissues and primary from metastatic melanomas in a microarray study on melanoma progression**. Expression data from the microarray analysis by Haqq et al [16] were collected for the genes identified in the RGP and Met libraries. The expression data for each gene group were submitted to SAM (FDR = 0) in a two-class analysis for detection of genes differentially expressed between primary and metastatic tumors and between non-neoplastic (skin and melanocytic nevi) and neoplastic (primary and metastatic melanomas) samples. The results from SAM analysis were extracted using SAMTERS and visualized by CLUSTER 3.0 and Java TreeView – Red and green squares represent genes up-regulated and down-regulated, respectively. (A) Expression profiles from primary and metastatic tumors for genes from the RGP library. (B – C) Expression profiles from non-neoplastic and neoplastic samples for genes from the RGP (B) and Met (C) libraries. Vertical blue lines on the left side indicate: (B) Genes from the RGP library that showed up-regulation in non-neoplastic samples; and (C) Genes from the Met library up-regulated in neoplastic tissues.

Since our validation results indicate that we have two collections of genes with truly differential expression between an RGP and a pool of metastatic melanoma cell lines, we decided to analyze the behavior of the subtracted genes in a panel of human melanoma tumors. We searched the data tables of a publicly available microarray study [16] that contains the expression profiles of samples from normal skin (3 samples), nevi (9 samples), primary (6 samples) and metastatic (19 samples) melanomas hybridized against a cDNA microarray containing 20,862 human cDNAs (representing 19,740 unique genes) from Research Genetics (Huntsville, AL). This work showed that metastatic melanomas exhibit two different gene expression signatures and that one of these signatures is shared with a sample from an RGP melanoma lesion. We were able to extract expression data for 194 genes of the RGP library and for 155 genes of the Met library. First, the set of data for each group was submitted to SAM (Significance Analysis of Microarray), setting the False Discovery Rate (FDR) to zero, in a two-class unpaired analysis where one group was represented by primary melanomas and the other group by metastatic melanomas. As shown in Fig. 5, a subset of RGP library genes was able to distinguish primary melanomas from metastasis, although two metastatic samples grouped with the primary tumors. A small group of genes from the RGP library were pointed as differentially expressed between primary and metastatic melanomas, including genes overexpressed in primary tumors as well as genes overexpressed in metastatic melanomas. The genes detected as down-regulated in most metastatic melanomas, and that are therefore candidates for metastasis suppressors, are *LUM *(lumican), *DCTN6 *(dynactin 6) and *DNCI2 *(dynein intermediate chain 2) (Fig. 5A). The Met library genes were not able to distinguish between primary and metastatic melanomas, since only one gene, *ENDOD1 *that codes for a putative endonuclease, was detected by SAM, at FDR = 0, as differentially expressed between the two tumor stages. In a second analysis, we performed SAM to compare non-neoplastic samples (skin and nevi) against tumor samples (primary and metastatic melanomas) for each set of genes (Fig. 5B and 5C). Genes from both libraries were found differentially expressed between non-neoplastic tissues and melanoma in both directions (up-regulated in non-neoplastic tissue/down-regulated in tumor, and down-regulated in non-neoplastic tissue/up-regulated in tumor). Since the WM1552C cells display markers of a less aggressive phenotype, for the RGP gene collection, we indicate a group of 14 genes (Fig. 5B, left side blue line) that were found to be consistently down-regulated in tumor samples. In contrast, a set of 14 Met library genes (Fig. 5C, left side blue line) showed a consistent up-regulation in tumor samples, compatible with an oncogenic role. The consistent differential expression profiles of these two groups of genes, distinguishing non-neoplastic from neoplastic tissues and primary melanomas from metastatic ones, make them good candidates for further studies in melanoma.

Treeview analysis of the genes validated by Northern blots showed that *DCN *is preferentially overexpressed in most skin and nevi samples and in 2 (out of 5) primary melanomas, confirming that its expression seems to decrease with melanoma progression, although a subset of melanoma samples also presented an overexpression of this gene (Fig. 5C, bottom). *MITF*, which was identified in the Met library, showed down-regulation in all skin samples, an increased expression in most nevi and overexpression in 11 out of 25 samples of primary and metastatic melanomas (Fig. 5C, bottom). The other two genes from the Met library (*PLP-1 *and *HLA-DRA*) did not exhibit expression pattern clearly associated to any specific stage of the melanoma progression in this study (Fig. 5C, bottom).

## Discussion

We report here the generation and analysis of two collections of subtracted cDNAs corresponding mostly to annotated mRNA. The collections are unique with only a few of the clones (2.5%) being common to both libraries, indicating that these collections represent a transcriptional content that distinguish between the RGP cells WM1552C and a group of four metastatic cell lines, and may reflect distinct transcriptional profiles of these two stages of melanoma progression (Additional File 2, Tables S1 and S2). Some of the sequences identified also contribute to extend annotated mRNA, as is the case for *ABCB5 *(Fig. S15), or reveal novel transcripts, since they match only genomic DNA, mapping in introns or intergenic regions (Additional File 3, Fig. S2–S14). We further assured that the subtraction process was efficient and validated the libraries by showing that all genes selected for validation by other methods confirmed the differential expression between the metastatic and the WM1552C cells (Fig. 3). Although some subtracted genes might reflect only individual genetic variations due to the use of a single cell line representing the RGP stage, we believe many of them truly represent cancer associated genes, since among them we have several known genes with characterized cancer related functions (Additional files 2 and 4).

### Potential metastasis suppressor pathways

Genes involved in DNA packaging, DNA repair and response to DNA damage are particularly enriched in the RGP library (Table 2), in accordance to the fact that early tumorigenic lesions have an activated DNA damage response and, in contrast to advanced tumors, are not marked by gross genomic instability [44,45]. The disproportion in the number of genes mapping to different chromosomes between the two libraries (Fig. 4) may be explained at least in part by chromosome abnormalities, which have been described for two of the cell lines used here, WM1552C (translocations involving 1p22, 5q34, 11p11, 12q11) and WM9 (loss of the long arm of chromosome 6 and gain of an extra copy of the entire chromosome 7) [7]. Since no extra copy of chromosome 1 occurs in WM1552C, the enrichment of chromosome 1 genes in the RGP library might be explained by losses involving this chromosome in the metastatic cell lines. Indeed, loss of the long arm and translocation involving chromosome 1 was observed in cell lines that are paired with the metastatic cell lines WM1617 and 1205Lu [6,46]. This is in agreement with the frequent rearrangements found in chromosome 1 in advanced melanomas [46-48].

Several of the genes identified in the RGP library have been reported as anti-tumorigenic or anti-metastatic, as for instance, *DCN *[43], *ANLN *[49], *HMGB2 *[50], *CXCL11 *[51]. Some of them are potentially involved in the KISS1 pathway, since *KISS1 *expression was detected only in WM1552C cells (Fig. 1). The *KISS1 *gene product, the secreted protein kisspeptin/metastin, plays an inhibitory role in chemotaxis and invasion of melanoma cells by a mechanism involving remodeling of the actin cytoskeleton [23]. Interestingly, many genes in the RGP library encode proteins associated to the actin cytoskeleton (see Additional File 2, Table S1 and Additional File 4, Table S3). Besides its role as a metastasis suppressor [20], first revealed in a melanoma model using SSH approach, kisspeptin/metastin and its receptor GPR54/KISS1R were recently implicated as important triggers of the complex process of sexual maturation [52,53]. The hormone leptin and its receptor also play important role in this process since leptin is a permissive factor for pubertal development [54], and the possibility that leptin modulates *KISS1 *expression in the central nervous system is under investigation [55]. In this context, the identification of *LEPR *gene, which codes for the leptin receptor, in the RGP library (Additional File 2, Table S1) might bear some relevance. Another gene with expression pattern similar to the one shown for *KISS1*, i.e., expressed exclusively in WM1552C (Fig. 3), is *DCN*. Both gene products, kisspeptin and decorin, besides having anti-migratory and anti-invasive roles in tumor cells are also implicated as regulators in the process of trophoblast invasion [56,57]. *LUM*, whose expression is down regulated in most metastatic melanomas (Fig. 5), is another member of the family of small proteoglycans that includes decorin for which an anti-invasive role was reported [58]. Therefore, novel genes with potential role in the maintenance of a status of low aggressiveness are likely to be represented in the RGP library, and some of them might be directly or indirectly associated to the *KISS1 *metastasis-suppressor pathway.

Of note, detection of *WNT5A *in the RGP library is in marked contrast to previous evidence that *WNT5A *is strongly associated with aggressiveness in human melanoma [59] and also to a most recent finding that *KISS1 *expression is down-regulated by Wnt5a [60]. This raises the interesting possibility that WM1552C cells carry an inactivating mutation that affects the Wnt5a signaling pathway.

Two of the RGP genes (*ALS2CR7 *and *MBOAT1*) validated by Northern blots encode proteins of unknown function, both highly expressed in WM1552C compared to VGP and metastatic cell lines making them interesting candidates for further investigations. This is the first evidence for the expression of these genes in melanocytic cells, and points towards a role in melanoma development.

### Potential oncogenic pathways

Among the genes represented in the Met library, many are associated to tumor growth, invasiveness and metastasis, such as *TM4SF1 *[61], *LAMA4 *[62], *G3BP2 *[63], *CD59 *antigen [64], and *SPP1 *[65]. Genes encoding proteins involved in the control of cell adhesion and cell migration are enriched in the Met library (Table 3). Among these genes, we have several growth factor receptors and integrins, including *ITGB8 *whose relevance in cancer remains to be characterized. Among the validated Met genes (*HLA-DRA*, *PLP1 *and *MITF*), several lines of evidence suggest their involvement in tumorigenesis. *HLA-DRA *encodes the α chain of the HLA-DR, one of the MHC class II molecules that, in contrast to MHC class I molecules, are not normally expressed by nonprofessional antigen-presenting cells (APC). Functional MHC class II molecules, key initiators of an immune response by activating CD4+ naïve T cells, are heterodimeric proteins composed of α and β chains encoded by separated genes (A and B). Melanocytes from normal skin and common nevus are negative for HLA class II molecules [66,67] while both primary and metastatic melanomas display heterogeneous levels of positive cells [67,68]. Although melanoma cells acquire HLA-DRA expression during tumor development, the prognostic value of this expression has not been clarified [68-73]. In contrast to the overexpression of HLA-DR α chain in several cancers, the HLA-DR β chain is not frequently overexpressed in cancer, suggesting that cancer cells do not express a functional HLA-DR receptor [74]. Clones corresponding to the *HLA-DRA *gene were the most abundantly sequenced clones from the Met library. However, sequences corresponding to the *HLA-DRB *gene were not detected in the same library, suggesting that HLA-DRA may not form a functional antigen-presenting molecule in these cells.

PLP1 is a transmembrane protein involved in myelinization [75] whose up regulation was detected in leiomyomas [76] and melanoma cell lines [77], and although no previous study addresses the role of *PLP1 *in melanoma development, remarkably, we show here that all vertical growth phase and metastatic cell lines exhibit high expression levels of this gene. It is interesting to note that *PLP1 *expression is regulated by the transcription factor SOX10 [78], which is also implicated in the regulation of *MITF *[79], the other validated gene.

*MITF *encodes a transcription factor required for melanocyte differentiation and survival [80,81] and we confirmed high expression levels of *MITF *mRNA in one of the metastatic cell lines, WM1617, which also showed the highest level of *PLP1 *expression. The high levels of *MITF *in WM1617 cells must explain the occurrence of many melanocytic markers (known MITF targets) in the Met library, as *TYRP1*, *TYR*, *DCT*, *MLANA*. However, differently from *PLP1*, high levels of *MITF *mRNA expression were not detected in the other three metastatic cell lines, but rather in cell lines from RGP and VGP tumor stages (WM35 and WM902). Consistent with this result, microarray studies revealed *MITF *overexpression in a subset of primary and metastatic melanoma samples [16,77] and led to the proposal [77] of a classification of melanoma cell lines independently of tumor stage. Interestingly, in the latter study, *PLP1*, *MITF *and the melanocytic markers were all detected as co-regulated genes associated to high proliferation and low metastatic potential in groups of cell lines that included WM1617 and WM35. So it is likely that a set of the genes detected in the Met library is more importantly linked to tumor proliferation/survival than invasion.

Although MITF is able to induce cell cycle arrest in melanocytes and melanoma cells in a p16 and p21 dependent manner [82,83], *MITF *gene was found to be amplified in melanoma and its overexpression induced transformation and drug resistance in *BRAF *mutant melanocytes [84]. In addition, recent works have identified as MITF transcriptional targets genes such as the *CDK2 *[85], the hipoxia induced factor *HIF1A *[86] and the hepatocyte growth factor receptor *MET *[87], all of them presenting functions that contribute to tumor development. Thus, the cellular circuits in which MITF is engaged are clearly complex and when and how MITF contributes to melanoma development are open questions. Since many of the known MITF targets are represented in the Met library, this collection may contain novel MITF targets and co-regulated genes whose identification will probably contribute to shed light on the MITF participation in melanoma development.

Other genes with consistent overexpression in tumor samples in comparison to nevi and skin (Fig. 5C) compatible with an oncogenic role include genes already associated to melanoma progression, such as *FN1*, which codes for the matrix protein fibronectin [88]; genes with proposed roles in tumor growth and metastasis, such as *ADAM10*, which encodes a putative desintegrin metallopeptidase [89], *SGK*, which encodes a glucocorticoid regulated kinase [90], *NRP2*, which encodes neuropilin 2[91], and also genes with no characterized function or association to cancer, such as *C18orf19*.

When we looked for the expression profile for RGP and Met genes in the microarray data obtained by Hoek et al [92], 13 and 19 genes, respectively, were found amongst the genes up-regulated in melanoma cell lines as compared to melanocytes (Additional File 5, Tables S5 and S6). Eleven genes (Additional File 6, Table S7) from our subtractive libraries were also detected as differentially expressed between murine tumorigenic melanoma cells and a parental nontumoral cell line in a study using proteomics and SAGE analysis [93]. Up-regulation of one of these genes, *NPM1 *(nucleophosmin), was found at both protein and transcript levels in melanoma cells, compatible with the detection of this gene in our Met library. An accumulation of a specific form of the nucleophosmin protein was also detected in human melanoma cell lines (including several of the WM cells used here) in comparison to normal melanocytes in another proteomics study [94].

## Conclusion

Altogether the data shown here strengthen previous evidence for several genes as candidate markers for melanoma progression and suggest that the subtractive libraries described are enriched in cancer-related genes, representing validated tools to be used in future studies for the identification of novel genes or pathways involved in melanoma progression.

## Competing interests

The author(s) declare that they have no competing interests.

## Authors' contributions

JFS designed the study, constructed, and sequenced the SSH libraries, conducted all expression validation and comparisons, and drafted the manuscript. EME conceived the study, participated in its design and coordination and also drafted and revised the manuscript. Both authors read and approved the final manuscript.

## Pre-publication history

The pre-publication history for this paper can be accessed here:



## Supplementary Material

Additional file 1 Representative image of agarose gels containing PCR-amplified inserts from 96 randomly selected clones from the subtractive libraries. The images show that most clones from both RGP and Met libraries carry inserts and most of them are ≥ 600 bp in length. Click here for file

Additional file 2 Complete lists of the genes identified in the RGP and Met libraries. The tables contain the identifier and annotation of the genes represented in the RGP and Met libraries and indicate the number of occurrences for each gene among the clones sequenced from each library. Click here for file

Additional file 3 Schematic representations of the sequence alignments of ESTs from the RGP and Met libraries that match intronic or intergenic regions. The data indicate that some of the sequences identified in the subtractive libraries may correspond to novel transcripts. Click here for file

Additional file 4 Lists of the genes represented by redundant clones in the RGP and Met libraries. The tables contain the identifier, Gene Ontology annotation and available information about role in cancer related processes for the genes represented by redundant clones in the RGP and Met libraries. Click here for file

Additional file 5 Search for the expression profile of the genes identified in the RGP and Met libraries in a melanoma microarray study performed by Hoek et al. [92]. Lists of the genes represented in the RGP and Met libraries that were detected as differentially expressed between melanocytes and melanoma cell lines in the above cited study. Click here for file

Additional file 6 Search for the expression profile of the genes identified in the RGP and Met libraries in the proteomics and SAGE analyses performed by de Souza et al. [93]. Lists of the genes represented in the RGP and Met libraries that were detected as differentially expressed between non-tumorigenic and tumorigenic murine melanocytic cell lines in the above cited study. Click here for file
