# Supplementary material for: Suppression subtractive hybridization profiles of radial growth phase and metastatic melanoma cell lines reveal novel potential targets
Source: BMC Cancer. 2008 Jan 22;8:19. doi: 10.1186/1471-2407-8-19 (PMC2267200; doi:10.1186/1471-2407-8-19)
Supplement: Additional file 1 — Representative image of agarose gels containing PCR-amplified inserts from 96 randomly selected clones from the subtractive libraries. The images show that most clones from both RGP and Met libraries carry inserts and most of them are ≥ 600 bp in length. [file 1471-2407-8-19-S1.pdf]

Additional File 1: Representative image of agarose gels containing PCR-amplified inserts from 96 randomly selected clones from the subtractive libraries. The images show that most clones from both RGP and Met libraries carry inserts and most of them are ≥ 600bp in length.

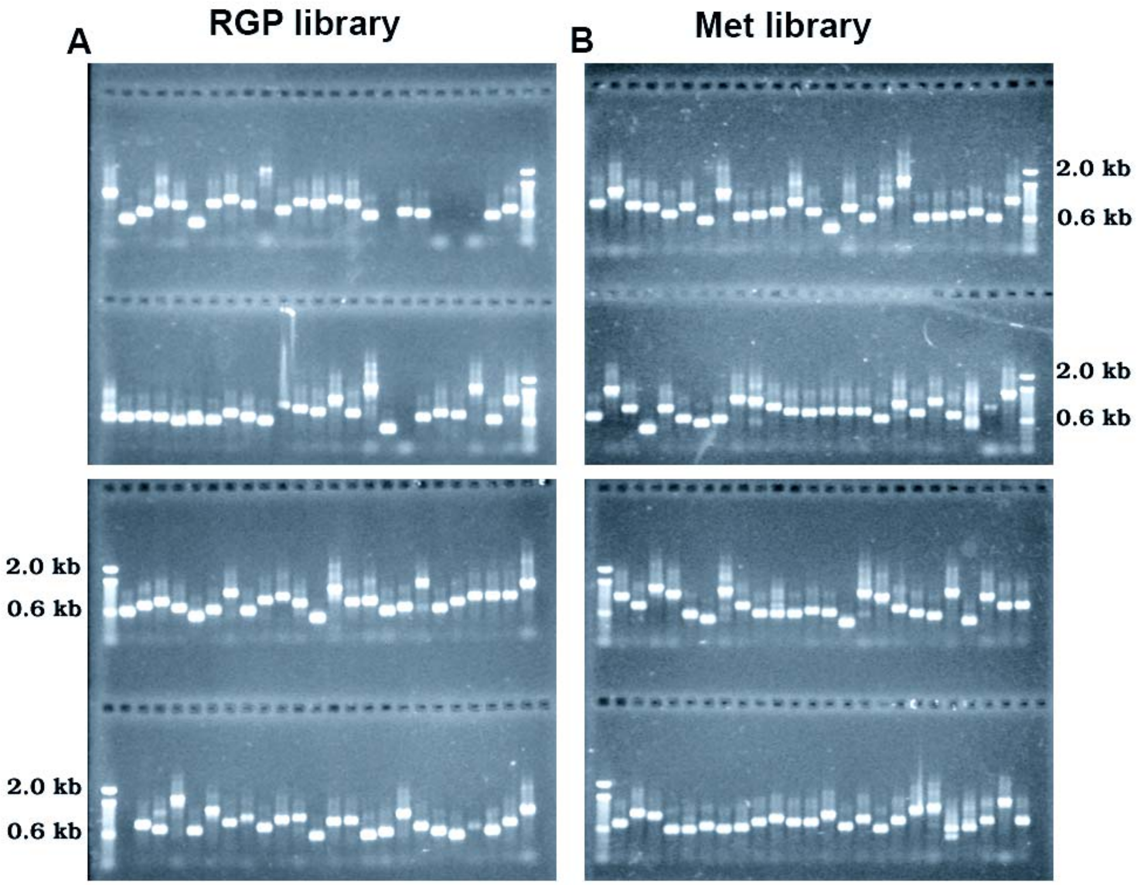

Figure S1: Most clones from both RGP and Met libraries carry inserts. Images from ethidium bromide stained agarose gels containing the PCR amplified inserts from 96 clones from the (A) RGP and (B) Met libraries. Molecular markers: 100 bp DNA ladder.
