# Supplementary material for: Suppression subtractive hybridization profiles of radial growth phase and metastatic melanoma cell lines reveal novel potential targets
Source: BMC Cancer. 2008 Jan 22;8:19. doi: 10.1186/1471-2407-8-19 (PMC2267200; doi:10.1186/1471-2407-8-19)
Supplement: Additional file 2 — Complete lists of the genes identified in the RGP and Met libraries. The tables contain the identifier and annotation of the genes represented in the RGP and Met libraries and indicate the number of occurrences for each gene among the clones sequenced from each library. [file 1471-2407-8-19-S2.pdf]

Additional File 2: Complete lists of the genes identified in the RGP and Met libraries. The tables contain the identifier, the annotations of the genes represented in the RGP and Met libraries and indicate the number of occurrences for each gene among the clones sequenced from each library.

Table S1: Genes represented in the RGP SSH library [a]

Gene	Gene Symbol	Accession number	Unigene	Chromosome	Redundancy
acetyl-Coenzyme A acetyltransferase 2	**ACAT2**	NM_005891	Hs.571037	chr6	2
actin, beta	**ACTB**	NM_001101	Hs.520640	chr7	3
actin, gamma 1	**ACTG1**	NM_001614	Hs.514581	chr17	2
actin-like 6A	**ACTL6A**	NM_004301	Hs.435326	chr3	1
AE(adipocyte enhancer)-binding protein 2	**AEBP2**	NM_153207	Hs.126497	chr12	1
A kinase (PRKA) anchor protein (yotiao) 9	**AKAP9**	NM_147166	Hs.651221	chr7	1
pyrroline-5-carboxylate synthetase	**ALDH18A1**	NM_002860	Hs.500645	chr10	1
aldehyde dehydrogenase 3 family, member A2	**ALDH3A2**	NM_000382	Hs.499886	chr17	1
aldehyde dehydrogenase 9 family, member A1	**ALDH9A1**	NM_000696	Hs.2533	chr1	1
amyotrophic lateral sclerosis 2 (juvenile) chromosome	**ALS2CR7**	NM_139158	Hs.348711	chr2	10
amphoterin induced gene 2	**AMIGO2**	NM_181847	Hs.121520	chr12	1
angiopoietin 1	**ANGPT1**	NM_001146	Hs.369675	chr8	1
anillin, actin binding protein (scraps homolog,Drosophila)	**ANLN**	NM_018685	Hs.62180	chr7	2
acidic (leucine-rich) nuclear phosphoprotein 32	**ANP32E**	NM_030920	Hs.603000	chr1	1
annexin A2	**ANXA2**	NM_004039	Hs.511605	chr15	1
adaptor-related protein complex 1, gamma 1 subunit	**AP1G1**	NM_001128	Hs.461253	chr16	1
Rho GTPase activating protein 18	**ARHGAP18**	NM_033515	Hs.486458	chr6	1
ubiquitin-conjugating enzyme E2 binding protein, 1 (Drosophila)	**ARIH1**	NM_005744	Hs.268787	chr15	1
ariadne homolog 2	**ARIH2**	NM_006321	Hs.31387	chr3	1
actin related protein 2/3 complex, subunit 3, 21kDa	**ARPC3**	NM_005719	Hs.524741	chr12	1
activating transcription factor 4 (tax-responsive enhancer element B67)	**ATF4**	NM_182810	Hs.496487	chr22	1
ATP synthase, H+ transporting, mit.l F0 complex, subunit F6	**ATP5J**	NM_001685	Hs.246310	chr21	1
alpha thalassemia/mental ret. syndrome X-linked(RAD54 h., S. cerevisiae)	**ATRX**	NM_000489	Hs.533526	chrX_	1
HLA-B associated transcript 1	**BAT1**	NM_004640	Hs.254042	chr6	1
bobby sox homolog	**BBX**	NM_020235	Hs.124366	chr3	1
Bcl-2-associated transcription factor	**BCLAF1**	NM_014739	Hs.486542	chr6	1
chromosome 12 open reading frame 4	**C12orf4**	NM_020374	Hs.302977	chr12	1
chromosome 13 open reading frame 3	**C13orf3**	NM_145061	Hs.88523	chr13	1
chromosome 14 open reading frame 108	**C14orf108**	NM_018229	Hs.528653	chr14	1
chromosome 14 open reading frame 112	**C14orf112**	NM_016468	Hs.137108	chr14	1
chromosome 14 open reading frame 2	**C14orf2**	NM_004894	Hs.109052	chr14	1
chromosome 15 open reading frame 41	**C15orf41**	NM_032499	Hs.48348	chr15	1
chromosome 1 open reading frame 103	**C1orf103**	NM_018372	Hs.25245	chr1	1
chromosome 21 open reading frame 6	**C21orf6**	NM_016940	Hs.34136	chr21	1
chromosome 4 open reading frame 27	**C4orf27**	NM_017867	Hs.406756	chr4	1

6 open reading frame 166	**C6orf166**	NM_018064	Hs.485915	chr6	1
chromosome 6 open reading frame 96	**C6orf96**	NM_017909	Hs.486835	chr6	1
calcium binding protein 39	**CAB39**	NM_016289	Hs.632536	chr2	1
adenylate cyclase-associated protein, 2	**CAP2**	AL832154	Hs.132902	chr6	1
calpain 2, (m/II) large subunit	**CAPN2**	NM_001748	Hs.350899	chr1	1
coiled-coil domain containing 80	**CCDC80**	NM_199511	Hs.477128	chr3	1
coiled-coil domain containing 82	**CCDC82**	NM_024725	Hs.525088	chr11	1
cyclin B1	**CCNB1**	NM_031966	Hs.23960	chr5	1
cyclin I	**CCNI**	NM_006835	Hs.648010	chr4	1
CD164 antigen, sialomucin	**CD164** [b]	NM_006016	Hs.520313	chr6	1RGP, 1MET
non-kinase Cdc42 effector protein SPEC2	**CDC42SE2**	NM_020240	Hs.508829	chr5	1
cell division cycle 73, Paf1/RNA polymerase II complex component, homolog	**CDC73**	NM_024529	Hs.576497	chr1	1
centromere protein N	**CENPN**	NM_018455	Hs.55028	chr16	1
H factor 1 (complement) (HF1)	**CFH**	NM_000186	Hs.363396	chr1	5
coiled-coil-helix-coiled-coil-helix domain containing 7	**CHCHD7**	NM_024300	Hs.436913	chr8	1
C-type lectin domain family 2, member B	**CLECSF2** [b]	NM_005127	Hs.85201	chr12	1RGP, 1MET
chloride intracellular channel 4	**CLIC4**	NM_013943	Hs.440544	chr1	1
cornichon homolog 4	**CNIH4**	NM_014184	Hs.445890	chr1	1
CCR4-NOT transcription complex, subunit 7	**CNOT7**	NM_054026	Hs.598387	chr8	2
coagulation factor C homolog, cochlin	**COCH**	NM_004086	Hs.21016	chr14	1
collagen, type XV, alpha 1	**COL15A1**	NM_001855	Hs.409034	chr9	1
CGI-120 protein	**COPZ1** [b]	NM_016057	Hs.505652	chr12	1RGP, 1MET
coronin, actin binding protein, 1C	**CORO1C**	NM_014325	Hs.637894	chr12	1
cytochrome c oxidase subunit VIIa polypeptide 2	**COX7A2**	NM_001865	Hs.70312	chr6	1
copine III	**CPNE3**	NM_003909	Hs.191219	chr8	1
cutA divalent cation tolerance homolog	**CUTA**	NM_015921	Hs.520070	chr6	1
chemokine (C-X-C motif) ligand 11	**CXCL11**	NM_005409	Hs.632592	chr4	2
DAZ associated protein 2	**DAZAP2**	NM_014764	Hs.369761	chr12	1
decorin	**DCN**	NM_133503	Hs.156316	chr12	8
DCP2 decapping enzyme homolog	**DCP2**	NM_152624	Hs.443875	chr5	1
dynactin 4 (p62)	**DCTN4**	NM_016221	Hs.328865	chr5	1
dynactin 6	**DCTN6**	NM_006571	Hs.158427	chr8	1
EST DKFZp781I0229	**DCUN1D1**	BX643780	Hs.104613	chr3	1
DEAD (Asp-Glu-Ala-Asp) box polypeptide 1	**DDX1**	NM_004939	Hs.440599	chr2	2
DEAD (Asp-Glu-Ala-Asp) box polypeptide 17	**DDX17**	NM_030881	Hs.528305	chr22	1
DEAD (Asp-Glu-Ala-Asp) box polypeptide 23	**DDX23**	NM_004818	Hs.130098	chr12	1
DEAD/H (Asp-Glu-Ala-Asp/His) box polypeptide 27	**DDX27**	NM_017895	Hs.65234	chr20	1
DEAD (Asp-Glu-Ala-Asp) box polypeptide 3, X-linked	**DDX3X**	NM_001356	Hs.380774	chrX	1
denticleless homolog	**DTL**	NM_016448	Hs.126774	chr1	1
DTW domain containing 1	**DTWD1**	NM_020234	Hs.127432	chr15	1
dynein, cytoplasmic, intermediate polypeptide 2	**DYNC1I2**	NM_001378	Hs.546250	chr2	1
epithelial cell transforming sequence 2 oncogene	**ECT2**	NM_018098	Hs.518299	chr3	1
clone IMAGE:3629020	**EIF4B**	BC013994	Hs.292063	chr12	1
cDNA: FLJ23586 fis	**EIF4E2**	AK027239	Hs.292026	chr2	1
eukaryotic translation initiation factor 4 gamma	**EIF4G2**	NM_001418	Hs.183684	chr11	2

ELAV (embryonic lethal, ab.vision, Drosolike(Hu antigen R)	**ELAVL1**	NM_001419	Hs.184492	chr19	1
ectonucleotide pyrophosphatase/phosphodiesterase 1	**ENPP1**	NM_006208	Hs.527295	chr6	1
family with sequence similarity 29	**FAM29A**	NM_017645	Hs.533468	chr9	1
cDNA FLJ31911 fis	**FAM33A**	AK056473	Hs.463607	chr17	2
family with sequence similarity 54	**FAM54A**	NM_138419	Hs.121536	chr6	2
family with sequence similarity 60	**FAM60A** [b]	NM_021238	Hs.505154	chr12	1RGP, 1MET
family with sequence similarity 62	**FAM62A**	NM_015292	Hs.632729	chr12	1
F-box only protein 5	**FBXO5**	NM_012177	Hs.645478	chr6	1
farnesyl diphosphate synthase	**FDPS**	NM_002004	Hs.335918	chr1	1
FGFR1 oncogene partner 2	**FGFR1OP2**	NM_015633	Hs.591162	chr12	1
hypothetical protein FLJ10292	**FLJ10292**	NM_018048	Hs.104650	chr12	1
hypothetical protein FLJ11184	**FLJ11184**	NM_018352	Hs.267446	chr4	1
hypothetical protein DKFZp566D234	**FSTL5**	NM_020116	Hs.591707	chr4	3
GATA binding protein 3	**GATA3**	NM_002051	Hs.524134	chr10	1
FLJ38642 fis	**GJA7**	AK095961	Hs.596755	chr17	1
glycosyltransferase AD-017	**GLT8D1**	NM_018446	Hs.297304	chr3	1
guanine nucleotide binding protein (G protein) gamma 11	**GNG11**	NM_004126	Hs.83381	chr7	2
full length insert cDNA clone EUROIMAGE 1674211	**GTF3C4**	AJ420430	Hs.22302	chr9	1
cDNA: FLJ22132 fis	**H2AFV**	AK025785	Hs.488189	chr7	1
H3 histone, family 3A	**H3F3A**	NM_002107	Hs.546259	chr2	1
histone acetyltransferase 1	**HAT1**	NM_003642	Hs.632532	chr2	1
histone deacetylase 1	**HDAC1**	NM_004964	Hs.88556	chr12	1
high-mobility group box 2	**HMGB2**	NM_002129	Hs.434953	chr4	2
cDNA: FLJ22083 fis,	**HMGCS1**	AK025736	Hs.397729	chr5	1
hypothetical protein FLJ10637	**HMGN3**	NM_018164	Hs.505077	chr12	1
heterogeneous nuclear ribonucleoprotein C (C1/C2)	**HNRPC**	NM_031314	Hs.508848	chr14	1
heterogeneous nuclear ribonucleoprotein U (scaffold attachment factor A)	**HNRPU**	AF068846	Hs.166463	chr1	1
heat shock protein 90kDa alpha (cytosolic), class A member 1	**HSP90AA1**	NM_001017963	Hs.525600	chr14	1
NAD(P) dependent steroid dehydrogenase-like	**HSPC105**	NM_145168	Hs.87779	chr16	1
MSTP028 protein	**KCTD10**	NM_031954	Hs.524731	chr12	1
potassium channel tetramerisation domain containing 3	**KCTD3**	NM_016121	Hs.335139	chr1	2
FLJ32145 fis	**KIAA0460**	AK056707	Hs.213666	chr1	1
kinesin family member 5B	**KIF5B**	NM_004521	Hs.591373	chr10	1
kinesin-associated protein 3	**KIFAP3**	NM_014970	Hs.433442	chr1	1
kelch-like 4 (Drosophila)	**KLHL4**	NM_019117	Hs.49075	chrX	1
lamin B receptor	**LBR**	NM_002296	Hs.435166	chr1	1
leptin receptor	**LEPR**	NM_002303	Hs.23581	chr1	1
hypothetical protein LOC643079	**LOC643079**	NM_001039896	Hs.591760	chr5	1
hypothetical protein FLJ36812	**LRRC57**	NM_153260	Hs.234681	chr15	1
LSM5 homolog, U6 small nuclear RNA associated (Scerevisiae)	**LSM5**	NM_012322	Hs.424908	chr7	1
latent transforming growth factor beta binding protein 1	**LTBP1**	NM_000627	Hs.49787	chr2	1
lumican	**LUM**	NM_002345	Hs.406475	chr12	1

melanoma antigen family C, 2	**MAGEC2**	NM_016249	Hs.123536	chrX	1
hypothetical protein MGC44669	**MBOAT1**	NM_175879	Hs.377830	chr6	4
malate dehydrogenase 1, NAD (soluble)	**MDH1**	NM_005917	Hs.526521	chr2	1
nuclear protein double minute 1	**MDM1**	NM_017440	Hs.591036	chr12	1
FLJ39869 fis	**MDM4**	AK097188	Hs.497492	chr1	1
microfibrillar-associated protein 4	**MFAP4**	NM_002404	Hs.296049	chr17	3
mitochondrial ribosomal protein L48	**MRPL48**	NM_016055	Hs.503239	chr11	1
c-myc binding protein	**MYCBP**	NM_012333	Hs.591506	chr1	2
MYST histone acetyltransferase 2	**MYST2**	NM_007067	Hs.21907	chr17	1
hypothetical protein DJ328E19.C1.1	**NBPF14** [b]	NM_015383	Hs.607640	chr1	2RGP, 1MET
neural precursor cell expressed, developmentally down-regulated 1	**NEDD1**	NM_152905	Hs.270084	chr12	1
NEFA-interacting nuclear protein NIP30	**NIP30**	NM_024946	Hs.396740	chr16	1
NME7	**NME7**	NM_013330	Hs.651246	chr1	2
non-POU domain containing, octamer-binding	**NONO**	NM_007363	Hs.533282	chrX	1
similar to rat nuclear ubiquitous casein kinase 2	**NUCKS1**	NM_022731	Hs.632458	chr1	2
nucleoporin 160kDa	**NUP160**	NM_015231	Hs.643526	chr11	3
nucleoporin Nup37	**NUP37**	NM_024057	Hs.444276	chr12	1
olfactory receptor, family 51, subfamily E, member 2	**OR51E2**	NM_030774	Hs.501758	chr21	2
origin recognition complex, subunit 2-like (yeast)	**ORC2L**	NM_006190	Hs.444870	chr2	1
oxysterol binding protein-like 8	**OSBPL8**	NM_020841	Hs.430849	chr12	1
OTU domain containing 6B	**OTUD6B**	NM_016023	Hs.30532	chr8	1
3-oxoacid CoA transferase	**OXCT1**	NM_000436	Hs.278277	chr5	1
hypothetical protein FLJ20604	**OXSM**	NM_017897	Hs.55781	chr3	1
platelet-activating factor acetylhydrolase	**PAFAH1B1**	NM_000430	Hs.77318	chr17	1
phosphoribosylaminoimidazole carboxylase	**PAICS**	NM_006452	Hs.518774	chr4	1
pre-B-cell colony-enhancing factor	**PBEF1** [b]	NM_005746	Hs.489615	chr7	1RGP, 1MET
dendritic cell protein	**PCID1**	NM_006360	Hs.502244	chr11	1
platelet derived growth factor C	**PDGFC**	NM_016205	Hs.570855	chr4	1
paternally expressed 10	**PEG10**	NM_015068	Hs.147492	chr7	1
PHD finger protein 17	**PHF17**	NM_024900	Hs.12420	chr4	1
PHD finger protein 20	**PHF20**	NM_016436	Hs.517044	chr20	2
phosphoinositide-3-kinase, class 3	**PIK3C3**	NM_002647	Hs.464971	chr18	1
pogo transposable element with ZNF domain	**POGZ**	NM_015100	Hs.591471	chr1	2
KIAA0039	**POLD3**	D26018	Hs.82502	chr11	2
polymerase (RNA) III (DNA directed) (62kD)	**POLR3C**	NM_006468	Hs.591457	chr1	1
protection of telomeres 1	**POT1**	NM_015450	Hs.31968	chr7	1
phosphoribosyl pyrophosphate amidotransferase	**PPAT**	NM_002703	Hs.331420	chr4	1
protein phosphatase 1, regulatory (inhibitor) subunit11	**PPP1R11**	NM_021959	Hs.82887	chr6	1
protein phosphatase 1, regulatory (inhibitor) subunit 12A	**PPP1R12A**	NM_002480	Hs.49582	chr12	1
phosphatase 2, regulatory subunit B (B56) gamma isoform	**PPP2R5C**	NM_178588	Hs.368264	chr14	1
palmitoyl-protein thioesterase 1	**PPT1**	NM_000310	Hs.3873	chr1	2
prolyl endopeptidase-like	**PREPL**	NM_001042385	Hs.444349	chr2	1
phosphoribosyl pyrophosphate synthetase 2	**PRPS2**	NM_002765	Hs.104123	chrX	1
pleckstrin homology, Sec7 and coiled-coil domains	**PSCDBP**	NM_004288	Hs.270	chr2	1

proteasome (prosome, macropain) subunit, alpha type, 4	**PSMA4**	NM_002789	Hs.251531	chr15	3
proteasome (prosome, macropain) subunit, alpha type, 5	**PSMA5**	NM_002790	Hs.485246	chr1	1
proteasome (prosome, macropain) subunit, beta type, 4	**PSMB4**	NM_002796	Hs.89545	chr1	1
proteasome (prosome, macropain) subunit, beta type, 6	**PSMB6**	NM_002798	Hs.77060	chr17	1
prostaglandin-endoperoxide synthase 2	**PTGS2**	NM_000963	Hs.196384	chr1	1
peroxisomal membrane protein 3, 35kDa	**PXMP3**	NM_000318	Hs.592781	chr8	1
RAD23 homolog B	**RAD23B**	NM_002874	Hs.521640	chr9	1
RAN, member RAS oncogene family	**RAN**	NM_006325	Hs.10842	chr6	1
karyopherin (importin) beta 3	**RANBP5**	NM_002271	Hs.643743	chr13	1
arginyl-tRNA synthetase-like	**RARSL**	NM_020320	Hs.485910	chr6	1
retinoblastoma binding protein 5	**RBBP5**	NM_005057	Hs.519230	chr1	1
RNA binding motif protein 25	**RBM25**	NM_021239	Hs.531106	chr14	2
RNA binding motif protein 34	**RBM34**	NM_015014	Hs.535224	chr1	1
chromosome condensation 1-like	**RCBTB2**	NM_001268	Hs.25447	chr13	1
v-rel reticuloendotheliosis viral oncogene homolog (avian)	**REL**	NM_002908	Hs.631886	chr2	1
regulator of G-protein signalling 2, 24kDa	**RGS2**	NM_002923	Hs.78944	chr1	2
ribosomal protein S27a	**RPS27A**	NM_002954	Hs.546292	chr2	1
RNA polymerase I transcription factor RRN3	**RRN3**	NM_018427	Hs.460078	chr16	1
runt-related transcription factor 2	**RUNX2**	NM_001015051	Hs.535845	chr6	2
secretogranin II (chromogranin C)	**SCG2**	NM_003469	Hs.516726	chr2	1
secretory granule, neuroendocrine protein 1 (7B2protein)	**SCG5**	NM_003020	Hs.156540	chr15	1
SEC24 related gene family, member C	**SEC24C**	NM_004922	Hs.81964	chr10	1
semaphorin 3A	**SEMA3A**	NM_006080	Hs.252451	chr7	1
SUMO-1-specific protease	**SENP6**	NM_015571	Hs.485784	chr6	1
Similar ao gene CDC10 cell division cycle 10 homolog	**SEPT7**	NM_001788	Hs.191346	chr7	1
SET translocation (myeloid leukemia-associated)	**SET**	NM_003011	Hs.436687	chr9	2
splicing factor 3a, subunit 3, 60kDa	**SF3A3**	NM_006802	Hs.77897	chr1	1
splicing factor 3b, subunit 1, 155kDa	**SF3B1**	NM_012433	Hs.632554	chr2	1
urvival of motor neuron protein interacting protein 1	**SIP1**	NM_003616	Hs.645400	chr14	1
solute carrier family 16 (monocarboxylic acidtransporters), member 1	**SLC16A1**	NM_003051	Hs.75231	chr1	1
solute carrier family 25 (mitochondrial carrier; phosphate carrier), member 24	**SLC25A24**	NM_013386	Hs.132553	chr1	1
solute carrier family 35, member B1	**SLC35B1**	NM_005827	Hs.154073	chr17	2
solute carrier family 38, member 2	**SLC38A2** [b]	NM_018976	Hs.221847	chr12	1RGP, 1MET
homolog of rat orphan transporter v7-3	**SLC6A15**	NM_182767	Hs.44424	chr12	1
homolog of C. elegans smu-1	**SMU1**	NM_018225	Hs.642743	chr9	1
SET and MYND domain containing 3	**SMYD3**	NM_022743	Hs.567571	chr1	1
small nuclear ribonucleoprotein polypeptide G	**SNRPG** [b]	NM_003096	Hs.516076	chr2	1RGP, 1MET
SRY (sex determining region Y)-box 4	**SOX4**	NM_003107	Hs.643910	chr6	1
synovial sarcoma translocation, chromosome 18	**SS18**	NM_005637	Hs.404263	chr18	1
signal sequence receptor, beta	**SSR2**	NM_003145	Hs.74564	chr1	1
stathmin 1/oncoprotein 18	**STMN1**	NM_005563	Hs.209983	chr1	1
activated RNA polymerase II transcription	**SUB1**	NM_006713	Hs.229641	chr5	1

cofactor 4					
NS1-associated protein 1	**SYNCRIP**	NM_006372	Hs.571177	chr6	1
SPT3-associated factor 42	**TADA1L**	NM_053053	Hs.435967	chr1	1
TAF12 RNA polymerase II, TATA box binding protein(TBP)-associated	**TAF12**	NM_005644	Hs.530251	chr1	1
hypothetical protein FLJ11046	**TBC1D23**	NM_018309	Hs.477003	chr3	1
transducin (beta)-like 1X-linked receptor 1	**TBL1XR1**	NM_024665	Hs.581171	chr3	1
transcription elongation factor A (SII)-like 8	**TCEAL8**	NM_001006684	Hs.389734	chrX	1
testis derived transcript (3 LIM domains)	**TES**	NM_152829	Hs.592286	chr7	1
putative MAPK activating protein (MGC3794)	**TIPRL**	NM_152902	Hs.209431	chr1	1
toll-like receptor 3	**TLR3**	NM_003265	Hs.543332	chr4	1
transmembrane protein 50B	**TMEM50B**	NM_006134	Hs.433668	chr21	1
hypothetical protein MAC30	**TMEM97**	NM_014573	Hs.199695	chr17	1
hypothetical protein FLJ90492	**TMTC3** [b]	NM_181783	Hs.331268	chr12	2RGP, 1MET
thiopurine S-methyltransferase	**TPMT**	NM_000367	Hs.444319	chr6	1
transcriptional intermediary factor 1	**TRIM24**	NM_003852	Hs.490287	chr7	1
transcription termination factor, RNA polymerase II	**TTF2**	NM_003594	Hs.486818	chr1	1
tRNA-yW synthesizing protein 3 homolog	**TYW3**	NM_138467	Hs.348411	chr1	1
UDP-N-acteylglucosamine pyrophosphorylase 1	**UAP1**	NM_003115	Hs.492859	chr1	1
ubiquitin-conjugating enzyme E2A (RAD6 homolog)	**UBE2A**	NM_003336	Hs.379466	chrX	1
ubiquitin-conjugating enzyme E2Q (putative)	**UBE2Q1**	NM_017582	Hs.607928	chr1	1
ubiquinol-cytochrome c reductase complex (7.2 kD)	**UCRC**	NM_013387	Hs.284292	chr22	1
pVHL-interacting deubiquitinating enzyme 1	**USP33**	NM_015017	Hs.480597	chr1	1
ubiquitin specific protease 8	**USP8**	NM_005154	Hs.644563	chr15	1
vacuolar protein sorting 33A (yeast)	**VPS33A**	NM_022916	Hs.592009	chr12	1
pumilio homolog 2 (Drosophila)	**VPS35**	NM_015317	Hs.467824	chr2	1
Wiskott-Aldrich syndrome protein interacting protein	**WASPIP**	NM_003387	Hs.591641	chr2	1
WD repeat membrane protein 19	**WDR19**	NM_025132	Hs.438482	chr4	1
WD repeat domain 35	**WDR35**	NM_001006657	Hs.205427	chr2	2
wingless-type MMTV integration site family, member 5A	**WNT5A**	NM_003392	Hs.643085	chr3	1
Ku autoantigen, 80kDa	**XRCC5**	NM_021141	Hs.388739	chr2	1
5'-3' exoribonuclease 2	**XRN2**	NM_012255	Hs.255932	chr20	1
xylosyltransferase I	**XYLT1**	NM_022166	Hs.22907	chr16	1
YME1-like 1 (S. cerevisiae) (YME1L1)	**YME1L1**	NM_139313	Hs.499145	chr10	1
tyrosine 3-monooxyg./tryp. 5-monooxyg.activ. prot, zeta polypeptide	**YWHAZ** [b]	NM_145690	Hs.492407	chr8	1RGP, 1MET
sterile alpha motif and leucine zipper containingkinase AZK	**ZAK**	NM_133646	Hs.444451	chr2	1
CCHC domain containing 9	**ZCCHC9**	NM_032280	Hs.15536	chr5	1
zinc finger, CDGSH-type domain 1	**ZCD1**	NM_018464	Hs.370102	chr10	1
zinc finger, DHHC domain containing 4	**ZDHHC4**	NM_018106	Hs.5268	chr7	1
zinc finger protein 146	**ZNF146**	NM_007145	Hs.643436	chr19	1
zinc finger protein 41	**ZNF41**	NM_153380	Hs.496074	chrX	1
NFX1-type containing 1	**ZNFX1**	NM_021035	Hs.371794	chr20	1
cDNA FLJ35976 fis		AK093295		chr1	1
cDNA IMAGE:4513453		BC039469	Hs.213061	chr1	1

cDNA IMAGE:5274919	BC033943	Hs.146268	chr1	1
cDNA RNA, FLJ21487	AK025140	Hs.552608	chr1	1
cDNA IMAGE:3028427	BC007568		chr1	1
cDNA DKFZp564O0122	AL049951	Hs.567380	chr1	1
EST	BX108371	Hs.201921	chr1	1
EST	BE613231	Hs.551847	chr1	1
EST	AF130080	Hs.213061	chr1	1
EST	AK025818	Hs.497575	chr1	1
EST	AK056963	Hs.193557	chr1	1
EST	AA863123	Hs.314413	chr13	1
EST	AA811365	Hs.648594	chr14	1
EST	AA811365	Hs.648594	chr14	1
cDNA DKFZp686D05115	BX537575	Hs.643531	chr16	1
cDNA clone IMAGE:4812621, partial cds	BC051737	Hs.597434	chr17	1
EST	DW462341	Hs.527989	chr17	1
EST	CR604143	Hs.644653	chr18	1
EST	BX482566	Hs.407368	chr19	1
EST	BU733606	Hs.572539	chr19	1
EST	AK094953	Hs.645225	chr19	1
EST	AK094953	Hs.645225	chr19	1
EST	CB857860	Hs.529272	chr2	1
EST	AA723885	Hs.516159	chr2	1
EST	BU740841	Hs.565756	chr21	1
cDNA clone IMAGE:4066387 5'	BF688069	Hs.307772	chr3	1
cDNA FLJ12839 fis	AK022901	Hs.636869	chr3	1
EST	AA248847	Hs.478000	chr3	1
cDNA clone IMAGE:4839074	BC047586	Hs.348292	chr5	1
cDNA FLJ14232	AK024294	Hs.114033	chr6	1
cDNA FLJ33993 fis	AK091312	Hs.408455	chr6	1
EST	DA435874	Hs.592692	chr6	1
EST	BF891265	Hs.519930	chr6	1
EST	BX105502	Hs.409578	chr6	1
EST	BQ371274		chr7	1
EST	DB351554	Hs.372541	chr7	1
EST	DB351554	Hs.372541	chr7	1
cDNA FLJ25402 fis	AK058131	Hs.104941	chr8	1
cDNA FLJ42399 fis	AK124390	Hs.592775	chr8	1
cDNA DKFZp686L07201	BX537532	Hs.355559	chrX	1

[a] Identification was done by BLAST or BLAT searches with the ESTs (deposited under GenBank accession numbers: ES315683-ES316049) obtained from RGP SSH library.

[b] Genes identified in both RGP and Met SSH libraries

Table S2: Genes represented in the Met SSH library [a]

Gene Name	Gene Symbol	Accession number	Unigene	Chromosome	Redundancy
alpha-2-macroglobulin	**A2M**	NM_000014	Hs.212838	chr12	12
ATP-binding cassette, sub-family B (MDR/TAP), member 5, mRNA (cDNA clone IMAGE:5539117), with apparent retained intron	**ABCB5**	NM_178559	Hs.404102	chr7	1
Acid phosphatase-like 2	**ACPL2**	NM_152282	Hs.255491	chr3	1
Activin A receptor, type IC	**ACVR1C**	NM_145259	Hs.352338	chr2	1
disintegrin and metalloproteinase domain 10 ADAM10	**ADAM10**	NM_001110	Hs.578508	chr15	1
alcohol dehydrogenase 5 (class III) chi polypeptide	**ADH5**	NM_000671	Hs.78989	chr4	1
aldehyde dehydrogenase 1 family, member A3	**ALDH1A3**	NM_000693	Hs.459538	chr15	1
apolipoprotein D	**APOD**	NM_001647	Hs.522555	chr3	2
Rho GTPase activating protein 15	**ARHGAP15**	NM_018460	Hs.171011	chr2	1
AT rich interactive domain 5B	**ARID5B**	NM_032199	Hs.535297	chr10	1
Activating signal cointegrator 1 complex subunit 1	**ASCC1**	NM_015947	Hs.500007	chr10	1
ATPase, H+ transporting, lysosomal 56/58kDa, V1	**ATP6V1B2**	NM_001693	Hs.295917	chr8	1
PTD012 protein (PTD012)	**C11orf54**	NM_014039	Hs.8360	chr11	1
Chromosome 18 open reading frame 19	**C18orf19**	NM_152352	Hs.13034	chr18	3
chromosome 3 open reading frame 1	**C3orf1**	NM_016589	Hs.477287	chr3	1
Ca2+-dependent activator protein for secretion 2	**CADPS2**	NM_017954	Hs.126730	chr7	1
calumenin	**CALU**	NM_001219	Hs.7753	chr7	1
CD164 antigen, sialomucin	**CD164** [b]	NM_006016	Hs.520313	chr6	1RGP, 1Met
antigen identified by monoclonal antibody MRC OX-2	**CD200**	NM_005944	Hs.79015	chr3	3
CD36 antigen (collagen type I receptor, thrombospondinreceptor)	**CD36**	NM_000072	Hs.120949	chr7	1
CD59 antigen p18-20	**CD59**	NM_000611	Hs.278573	chr11	9
chitinase 3-like 2	**CHI3L2**	NM_004000	Hs.514840	chr1	2
C-type lectin domain family 2, member B	**CLECSF2** [b]	NM_005127	Hs.85201	chr12	1RGP, 1Met
collectin sub-family member 10 (C-type lectin)	**COLEC10**	NM_006438	Hs.176615	chr8	1
Coatomer protein complex, subunit zeta 1	**COPZ1** [b]	NM_016057	Hs.505652	chr12	1
carboxypeptidase D	**CPD**	NM_001304	Hs.446079	chr17	1
cytoplasmic polyadenylation element binding protein 4	**CPEB4**	NM_030627	Hs.127126	chr5	1
carboxypeptidase M	**CPM**	NM_001005502	Hs.484551	chr12	2
carboxypeptidase, vitellogenic-like	**CPVL**	NM_019029	Hs.233389	chr7	1
cartilage associated protein	**CRTAP**	NM_006371	Hs.517888	chr3	1
connective tissue growth factor	**CTGF**	U14750	Hs.591346	chr6	1
catenin (cadherin-associated protein), beta 1, 88kDa	**CTNNB1**	NM_001904	Hs.476018	chr3	1
catenin (cadherin-associated protein), delta 1	**CTNND1**	NM_001331	Hs.166011	chr11	1
cathepsin K (pycnodysostosis)	**CTSK**	NM_000396	Hs.632466	chr1	2
cullin 5	**CUL5**	NM_003478	Hs.440320	chr11	1
cylindromatosis (turban tumor syndrome)	**CYLD**	NM_015247	Hs.578973	chr16	1
dopachrome tautomerase (dopachrome delta-isomerase tyrosine-related protein 2)	**DCT**	NM_001922	Hs.301865	chr13	6
DEAD (Asp-Glu-Ala-Asp) box polypeptide 5	**DDX5**	NM_004396	Hs.279806	chr17	1

DEAD/H (Asp-Glu-Ala-Asp/His) box polypeptide 8 (RNAhelicase)	**DHX8**	NM_004941	Hs.463105	chr17	1
dynein, light chain, Tctex-type 3	**DYNLT3**	NM_006520	Hs.446392	chrX	1
E3 ubiquitin protein ligase, HECT domain containing, 1	**EDD1**	NM_015902	Hs.591856	chr8	1
eukaryotic translation initiation factor 1A, Y chromosome	**EIF1AY**	NM_004681	Hs.461178	chrY	1
KIAA0830 protein	**ENDOD1**	BC026191	Hs.167115	chr11	1
EphA3	**EPHA3**	NM_005233	Hs.123642	chr3	1
SEC10-like 1 (S. cerevisiae)	**EXOC5**	NM_006544	Hs.334936	chr14	1
fatty acid binding protein 7, brain	**FABP7**	NM_001446	Hs.26770	chr6	1
Family with sequence similarity 60, member A	**FAM60A** [b]	NM_021238	Hs.505154	chr12	1RGP, 1Met
cDNA DKFZp586I0521	**FAM80B**	AL137567	Hs.504670	chr12	1
Family with sequence similarity 82, member B	**FAM82B**	AK000672	Hs.145386	chr8	1
Fc receptor homolog expressed in B cells	**FCRLM1**	NM_032738	Hs.266331	chr1	1
Farnesyl-diphosphate farnesyltransferase 1	**FDFT1**	NM_004462.3	Hs.593928	chr8	1
hypothetical protein LOC392636	**FLJ16237**	NM_001004320	Hs.155348	chr7	1
fibronectin 1	**FN1**	NM_002026	Hs.203717	chr2	1
frizzled homolog 1 (Drosophila)	**FZD1**	NM_003505	Hs.94234	chr7	1
Ras-GTPase activating protein SH3 domain-bindingprotein 2	**G3BP2**	NM_012297	Hs.303676	chr4	3
cDNA UGPQ mRNA, complete cds	**GDPD1**	AY271346	Hs.631744	chr17	1
Glycoprotein M6B	**GPM6B**	NM_001001994	Hs.495710	chrX	3
Homo sapiens G protein-coupled receptor 126	**GPR126**	NM_020455	Hs.318894	chr6	1
G-rich RNA sequence binding factor 1	**GRSF1**	NM_002092	Hs.309763	chr4	1
general transcription factor IIH	**GTF2H1**	NM_005316	Hs.577202	chr11	1
major histocompatibility complex, class II	**HLA-DRA**	NM_019111	Hs.520048	chr6	55
hemicentin	**HMCN1**	NM_031935	Hs.58877	chr1	1
high-mobility group box 1	**HMGB1**	NM_002128	Hs.434102	chr13	1
Hermansky-Pudlak syndrome 5	**HPS5**	NM_181507	Hs.437599	chr11	1
heat shock 90kDa protein 1, beta	**HSP90AB1**	NM_007355	Hs.509736	chr6	1
shock 70kDa protein 5 (glucose-regulated protein78kDa)	**HSPA5**	NM_005347	Hs.605502	chr9	1
insulin-degrading enzyme	**IDE**	NM_004969	Hs.500546	chr10	1
interferon-induced protein with tetratricopeptide repeats 1	**IFIT1**	NM_001548	Hs.20315	chr10	1
interleukin 1 receptor accessory protein	**IL1RAP**	NM_002182	Hs.478673	chr3	1
interleukin 6 (interferon, beta 2)	**IL6**	NM_000600	Hs.512234	chr7	1
interleukin enhancer binding factor 2, 45kDa	**ILF2**	NM_004515	Hs.75117	chr1	1
inositol(myo)-1(or 4)-monophosphatase 1	**IMPA1**	NM_005536	Hs.555086	chr8	1
integrin, alpha 6	**ITGA6**	NM_000210	Hs.133397	chr2	1
integrin, beta 1 (fibronectin receptor)	**ITGB1**	NM_133376	Hs.643813	chr10	1
Integrin, beta 8	**ITGB8**	NM_002214	Hs.592171	chr7	4
inositol 1,4,5-triphosphate receptor, type 1	**ITPR1**	NM_002222	Hs.567295	chr3	3
cDNA clone IMAGE:5312122	**KIAA0738**	BC039457	Hs.406492	chr7	1
KIAA1815	**KIAA1815**	NM_024896.2	Hs.591078	chr9	1
cDNA clone CS0DF027YK02	**KIF2**	CR601526	Hs.558351	chr5	1
Kelch-like 9 (Drosophila)	**KLHL9**	NM_018847	Hs.522029	chr9	1
Karyopherin alpha 1	**KPNA1**	NM_002264.2	Hs.161008	chr3	1
laminin, alpha 4	**LAMA4**	NM_002290	Hs.213861	chr6	2
lysosomal-associated membrane protein 2	**LAMP2**	NM_002294	Hs.496684	chrX	1

cDNA DKFZp686P07116, proximo gene PYGO1	**LOC283658**	AL833463	Hs.87194	chr15	1
EUROIMAGE 2005635, próximo do mir146a	**LOC285628**	AL389942	Hs.604728	chr5	3
Similar to RIKEN cDNA 2310016C16	**LOC493869**	NM_001008397.1	Hs.289044	chr5	1
cDNA EUROIMAGE 588495, mRNA	**LOC58489**	BC059401	Hs.459072	chr15	1
cDNA clone IMAGE:5578073	**LOC643401**	BC039509	Hs.533212	chr5	1
loss of heterozygosity, 11, chromosomal region 2, geneA	**LOH11CR2A**	NM_014622	Hs.152944	chr11	1
low density lipoprotein-related protein 2	**LRP2**	NM_004525	Hs.470538	chr2	1
LTV1 homolog (S. cerevisiae)	**LTV1**	NM_032860	Hs.185675	chr6	1
Mitogen-activated protein kinase 1	**MAPK1**	NM_002745	Hs.431850	chr22	1
muscleblind-like 2 (Drosophila)	**MBNL2**	NM_144778	Hs.134221	chr13	1
matrix Gla protein	**MGP**	NM_000900	Hs.365706	chr12	1
Mesoderm induction early response 1, family member 3	**MIER3**	NM_152622.3	Hs.657594	chr5	1
microphthalmia-associated transcription factor	**MITF**	NM_000248	Hs.166017	chr3	1
melan-A	**MLANA**	NM_005511	Hs.154069	chr9	2
myeloid/lymphoid or mixed-lineage leukemia3	**MLL3**	NM_170606	Hs.647120	chr7	1
matrix metalloproteinase 8 (neutrophil collagenase)	**MMP8**	NM_002424	Hs.161839	chr11	1
mitochondrial ribosomal protein L32	**MRPL32**	NM_031903	Hs.50252	chr7	1
mitochondrial ribosomal protein L42	**MRPL42**	NM_172178	Hs.199579	chr12	2
Metadherin	**MTDH**	NM_178812	Hs.377155	chr8	1
myotubularin related protein 6	**MTMR6**	NM_004685	Hs.643702	chr13	1
mitochondrial tumor suppressor gene 1	**MTUS1**	NM_020749	Hs.7946	chr8	3
Neuroblastoma breakpoint family, member 14	**NBPF14** [b]	NM_015383	Hs.607640	chr1	1RGP, 1Met
nuclear receptor coactivator 4	**NCOA4**	NM_005437	Hs.643658	chr10	1
Nuclear factor I/B	**NFIB**	NM_005596.2	Hs.370359	chr9	1
NF-kappa B-repressing factor	**NKRF**	NM_017544	Hs.437084	chrX	1
nucleophosmin (nucleolar phosphoprotein B23)	**NPM1**	NM_002520	Hs.557550	chr5	1
neuronal cell adhesion molecule	**NRCAM**	NM_005010	Hs.21422	chr7	1
Neuropilin 2	**NRP2**	NM_003872	Hs.471200	chr2	2
Neurexin 3	**NRXN3**	NM_004796	Hs.368307	chr14	1
origin recognition complex, subunit 4-like	**ORC4L**	NM_002552	Hs.558364	chr2	1
oxidase (cytochrome c) assembly 1-like	**OXA1L**	NM_005015	Hs.151134	chr14	1
purinergic receptor P2Y, G-protein coupled, 5	**P2RY5**	NM_005767	Hs.123464	chr13	1
poly(A) binding protein, cytoplasmic 1	**PABPC1**	NM_002568	Hs.387804	chr8	1
Phosphoprotein associated with glycosphingolipid microdomains 1	**PAG1**	NM_018440.3	Hs.266175	chr8	1
pre-B-cell colony-enhancing factor	**PBEF1** [b]	NM_005746	Hs.489615	chr7	1RGP, 1Met
platelet-derived growth factor receptor,alpha	**PDGFRA**	NM_006206	Hs.74615	chr4	2
peroxisomal biogenesis factor 3	**PEX3**	NM_003630	Hs.7277	chr6	1
phosphoglycerate mutase 1 (brain)	**PGAM1**	NM_002629	Hs.632918	chr12	1
proteolipid protein 1	**PLP1**	NM_000533	Hs.1787	chrX	24
Chromosome 13 open reading frame 12	**POMP**	NM_015932	Hs.268742	chr13	2
Protein phosphatase 2C, magnesium-dependent, catalytic subunit	**PPM2C**	NM_018444	Hs.22265	chr8	1
proteoglycan 1, secretory granule	**PRG1**	NM_002727	Hs.1908	chr10	1
prion protein (p27-30)	**PRNP**	NM_183079	Hs.472010	chr20	1
proteasome (prosome, macropain) subunit, alpha type, 1	**PSMA1**	NM_148976	Hs.102798	chr11	1

proteasome (prosome, macropain) 26S subunit	**PSMD12**	NM_174871	Hs.646575	chr17	1
protein tyrosine phosphatase, non-receptor type 3	**PTPN3**	NM_002829	Hs.436429	chr9	1
retinoic acid induced 14	**RAI14**	NM_015577	Hs.431400	chr5	1
retinoic acid receptor, beta	**RARB**	NM_016152	Hs.536687	chr3	1
retinoblastoma 1 (including osteosarcoma)	**RB1**	NM_000321	Hs.408528	chr13	1
RNA binding motif protein, X-linked 2	**RBMX2**	NM_016024	Hs.61184	chrX	1
radixin	**RDX**	NM_002906	Hs.263671	chr11	2
cDNA clone MGC:24463 IMAGE:4082362, mRNA	**REEP3**	BC018658	Hs.499833	chr10	1
ring finger protein 111	**RNF111**	NM_017610	Hs.404423	chr15	1
ribosomal protein L36a	**RPL36A**	NM_021029	Hs.432485	chrX	1
ribosomal protein S13	**RPS13**	NM_001017	Hs.446588	chr11	1
ribosomal protein S4, Y-linked	**RPS4Y1**	NM_001008	Hs.282376	chrY	1
Remodeling and spacing factor 1	**RSF1**	NM_016578	Hs.420229	chr11	1
scrapie responsive protein 1	**SCRG1**	NM_007281	Hs.7122	chr4	1
KIAA0193 gene product	**SCRN1**	NM_014766	Hs.520740	chr7	1
SDA1 domain containing 1	**SDAD1**	NM_018115	Hs.632604	chr4	1
selenoprotein K	**SELK**	NM_021237	Hs.58471	chr3	1
serine (or cysteine) proteinase inhibitor	**SERPINE2**	NM_006216	Hs.38449	chr2	4
secreted frizzled-related protein 1	**SFRP1**	NM_003012	Hs.213424	chr8	1
splicing factor, arginine/serine-rich 2	**SFRS2**	NM_003016	Hs.584801	chr17	1
serum/glucocorticoid regulated kinase	**SGK**	NM_005627	Hs.510078	chr6	2
solute carrier family 35, member A5	**SLC35A5**	NM_017945	Hs.237480	chr3	1
solute carrier family 38, member 2	**SLC38A2** [b]	NM_018976	Hs.221847	chr12	1RGP, 1Met
Solute carrier family 39 (zinc transporter), member 9	**SLC39A9**	NM_018375	Hs.432690	chr14	1
solute carrier family 5 (low affinity glucose cotransporter), member 4	**SLC5A4**	NM_014227	Hs.130101	chr22	2
small nuclear ribonucleoprotein polypeptide B	**SNRPB2**	NM_003092	Hs.280378	chr20	1
small nuclear ribonucleoprotein polypeptide G	**SNRPG** [b]	NM_003096	Hs.516076	chr2	1RGP, 1Met
Spermatogenesis associated, serine-rich 1	**SPATS1**	NM_145026	Hs.135283	chr6	1
secreted phosphoprotein 1 (osteopontin)	**SPP1**	NM_000582	Hs.313	chr4	2
sprouty-related, EVH1 domain containing 1	**SPRED1**	NM_152594	Hs.525781	chr15	1
sprouty homolog 4 (Drosophila)	**SPRY4**	NM_030964	Hs.323308	chr5	1
sperm specific antigen 2	**SSFA2**	NM_006751	Hs.591602	chr2	1
synaptotagmin-like 2	**SYTL2**	NM_032943	Hs.369520	chr11	1
tryptophan 2,3-dioxygenase	**TDO2**	NM_005651	Hs.183671	chr4	7
transferrin	**TF**	NM_001063	Hs.518267	chr3	1
transforming growth factor, beta-induced, 68kDa	**TGFBI**	NM_000358	Hs.369397	chr5	1
thrombospondin 2	**THBS2**	NM_003247	Hs.371147	chr6	1
tissue inhibitor of metalloproteinase 3	**TIMP3**	NM_000362	Hs.644633	chr22	1
transmembrane 4 superfamily member 1	**TM4SF1**	NM_014220	Hs.351316	chr3	10
Transmembrane protein 100	**TMEM100**	NM_018286	Hs.173233	chr17	1
Transmembrane protein 131	**TMEM131**	BC036841	Hs.469376	chr2	1
transmembrane protein 2 (TMEM2)	**TMEM2**	NM_013390	Hs.494146	chr9	1
Transmembrane and tetratricopeptide repeat containing 3	**TMTC3** [b]	CR749309	Hs.331268	chr12	1RGP, 1Met
tumor necrosis factor, alpha-induced protein 6	**TNFAIP6**	NM_007115	Hs.437322	chr2	1
Tumor necrosis factor superfamily, member 5-induced protein 1	**TNFSF5IP1**	NM_020232	Hs.464652	chr18	1
thiamin pyrophosphokinase 1	**TPK1**	NM_022445	Hs.490454	chr10	1

Tripartite motif-containing 33	**TRIM33**	NM_015906	Hs.26837	chr1	1
tumor susceptibility gene 101	**TSG101**	NM_006292	Hs.523512	chr11	1
tyrosinase (oculocutaneous albinism IA)	**TYR**	NM_000372	Hs.503555	chr11	2
tyrosinase-related protein 1	**TYRP1**	NM_000550	Hs.270279	chr9	2
UDP-glucose ceramide glucosyltransferase-like 2	**UGCGL2**	NM_020121	Hs.193226	chr13	1
UDP-glucose pyrophosphorylase 2	**UGP2**	NM_006759	Hs.516217	chr2	1
UDP glycosyltransferase 8 (UDP-galactose ceramidegalactosyltransferase)	**UGT8**	NM_003360	Hs.144197	chr4	1
ubiquinol-cytochrome c reductase hinge protein	**UQCRH**	NM_006004	Hs.481571	chr1	1
Chromosome 14 open reading frame 150	**WDR89**	NM_080666	Hs.509585	chr14	1
tyrosine 3-monooxyg./tryp. 5-monooxyg.activ. prot, zeta polypeptide	**YWHAZ** [b]	NM_145690	Hs.492407	chr8	1RGP, 1Met
cDNA FLJ90443 fis	**ZC3H13**	AK074924	Hs.136102	chr13	1
Zinc finger protein 512	**ZNF512**	NM_032434.2	Hs.529178	chr2	1
EST		BQ437417	Hs.633116	chr1	1
clone IMAGE:5019705 extremidade 5' do SIDT2 ou 3'doPAFAH1B2		BC021287	Hs.410977	chr11	1
EST		BC037984	Hs.91791	chr11	1
cDNA FLJ31578 fis, clone NT2RI2001952		AK056140	Hs.505983	chr12	1
EST		AA012878	Hs.434604	chr12	1
EST		BE799296	Hs.434604	chr12	1
EST		BX382682	Hs.199579	chr12	1
EST		AI831849	Hs.271014	chr12	1
cDNA deleted in lymphocytic leukemia, 2 (DLEU2)		NM_006021	Hs.547964	chr13	1
EST		BF343525	Hs.134687	chr15	1
cDNA hypothetical protein LOC146481, mRNA (cDNA clone IMAGE:4639919), partial cds		BC023607	Hs.316564	chr16	1
cDNA FLJ26484 fis		AK129994	Hs.447555	chr17	1
EST		DA506880		chr17	1
cDNA FLJ25631 fis		AK098497	Hs.464416	chr18	1
EST		AI610957	Hs.594083	chr18	1
EST		G36677		chr18	1
cDNA hypothetical protein FLJ37953		NM_001039693	Hs.204619	chr2	1
cDNA: FLJ22105 fis, próximo gene NFATC2		AK025758	Hs.356321	chr20	1
EST		AX750578		chr21	1
cDNA, IMAGE:5278517, extremidade 5'do gene NR2C2		BC039246	Hs.651299	chr3	1
cDNA clone IMAGE:3919084tissue_type="Skin, melanotic melanoma		BC028978	Hs.374715	chr5	1
EST		AA046162		chr5	1
EST		BU183727		chr5	1
EST		U52054	Hs.561411	chr5	1
EST		DA293172	Hs.277154	chr5	1
EST		CN292792	Hs.533212	chr5	1
cDNA IMAGE:4706427, intron do C7orf10		BC016631	Hs.586313	chr7	1
cDNA AF056184		AF056184		chr7	1
EST		BM712473	Hs.7753	chr7	1
EST		BG110543	Hs.133107	chr8	1
EST		BE142170		chrX	1

[a] Identification was done by BLAST or BLAT searches with the ESTs (deposited under GenBank accession numbers: ES316050-ES316435) obtained from Met SSH library.

[b] Genes identified in both RGP and Met SSH libraries
