# Supplementary material for: Suppression subtractive hybridization profiles of radial growth phase and metastatic melanoma cell lines reveal novel potential targets
Source: BMC Cancer. 2008 Jan 22;8:19. doi: 10.1186/1471-2407-8-19 (PMC2267200; doi:10.1186/1471-2407-8-19)
Supplement: Additional file 3 — Schematic representations of the sequence alignments of ESTs from the RGP and Met libraries that match intronic or intergenic regions. The data indicate that some of the sequences identified in the subtractive libraries may correspond to novel transcripts. [file 1471-2407-8-19-S3.pdf]

**Additional File 3:** Schematic representations of the sequence alignments of ESTs from the RGP and Met libraries that match intronic or intergenic regions. The data indicate that some of the sequences identified in the subtractive libraries may correspond to novel transcripts.

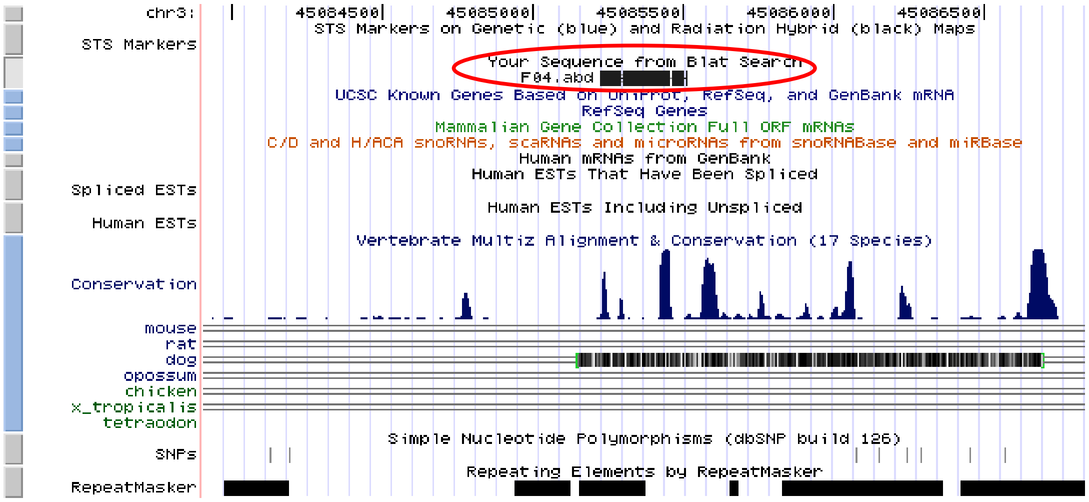


**Figure S2: Schematic view of the sequence alignment for an EST (GenBank accession number ES315792) from the RGP library that matches only an intergenic region on human chromosome 3.** Using the BLAT tool (http://genome.ucsc.edu/cgi-bin/hgBlat), the sequence (represented in the red ellipse) was aligned to the human genome and the original scheme generated by the BLAT tool was downloaded and displayed here.

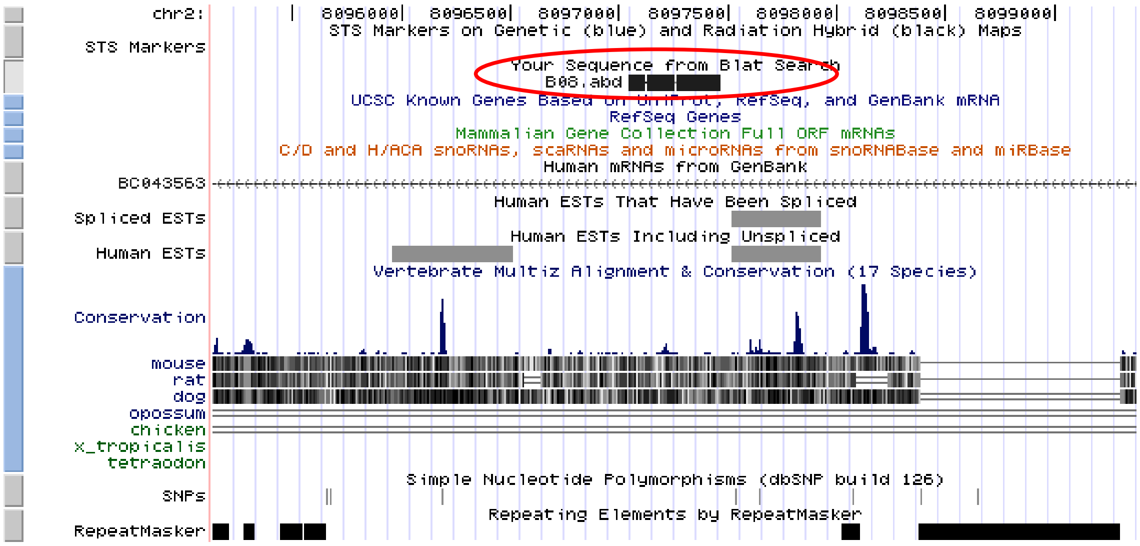

**Figure S3: Schematic view of the sequence alignment for an EST (GenBank accession number ES315760) from the RGP library that matches only an intergenic region on human chromosome 2.** Using the BLAT tool (http://genome.ucsc.edu/cgi-bin/hgBlat), the sequence (represented in the red ellipse) was aligned to the human genome and the original scheme generated by the BLAT tool was downloaded and displayed here.

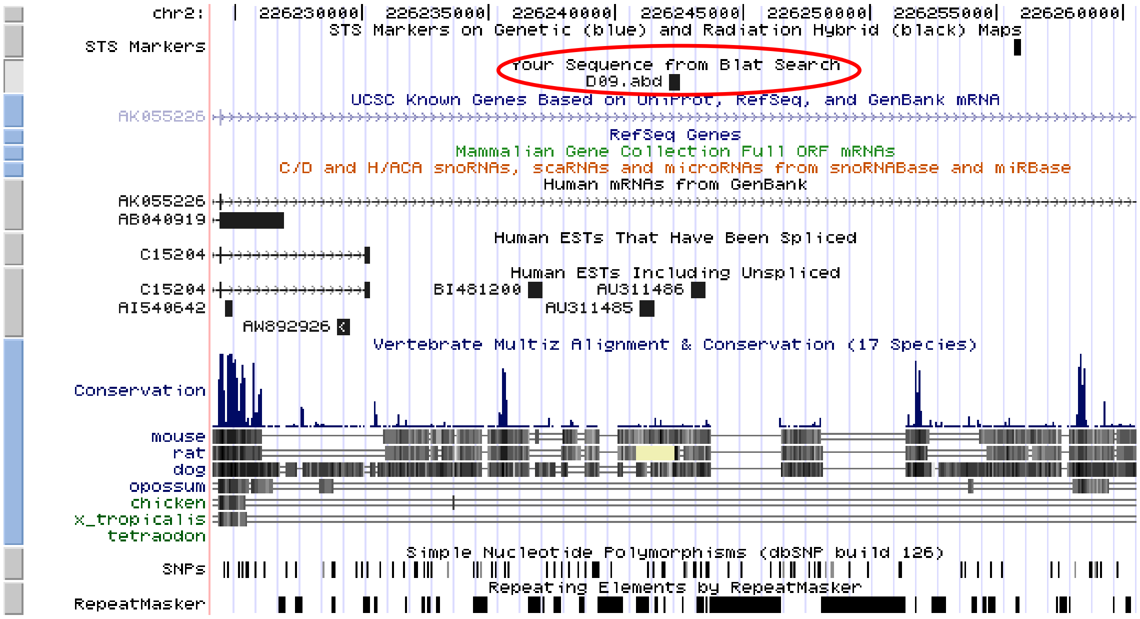

**Figure S4: Schematic view of the sequence alignment for an EST (GenBank accession number ES315900) from the RGP library that matches an intronic region (cDNA AK055226) on human chromosome 2.** Using the BLAT tool (http://genome.ucsc.edu/cgi-bin/hgBlat), the sequence (represented in the red ellipse) was aligned to the human genome and the original scheme generated by the BLAT tool was downloaded and displayed here.

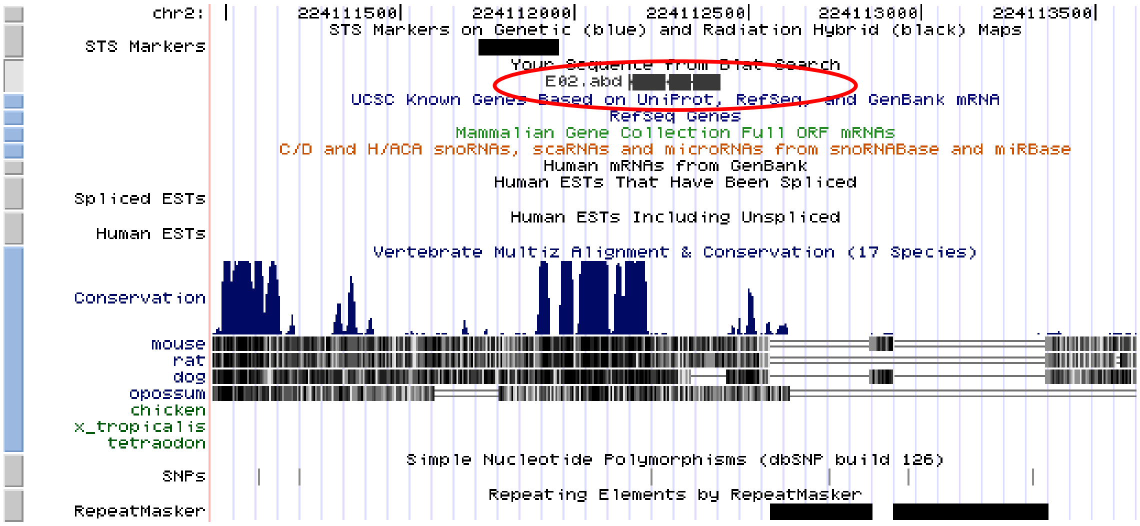


**Figure S5: Schematic view of the sequence alignment for an EST (GenBank accession number ES315904) from the RGP library that matches only an intergenic region on human chromosome 2.** Using the BLAT tool (http://genome.ucsc.edu/cgi-bin/hgBlat), the sequence (represented in the red ellipse) was aligned to the human genome and the original scheme generated by the BLAT tool was downloaded and displayed here.

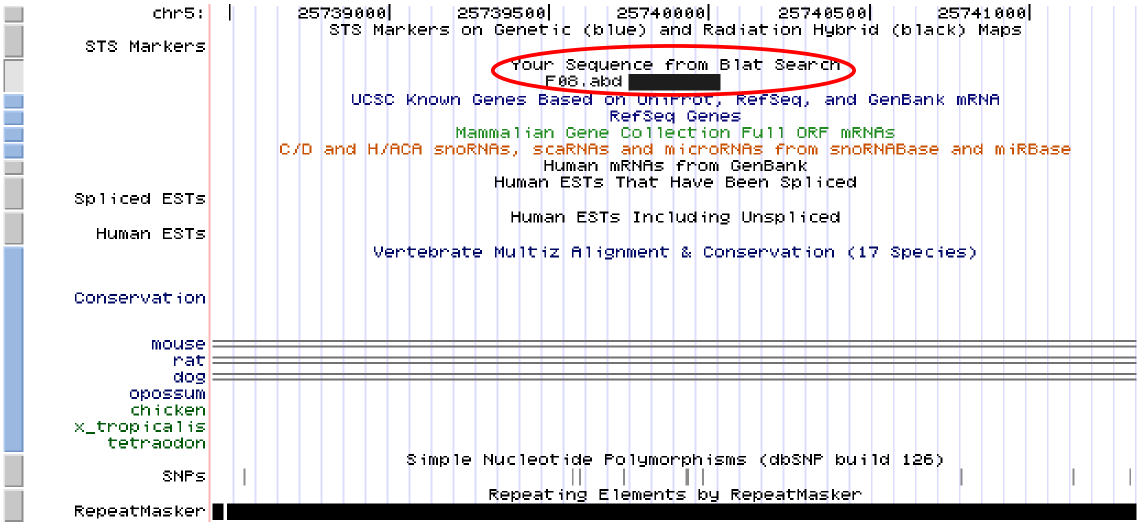


**Figure S6: Schematic view of the sequence alignment for an EST (GenBank accession number ES315721) from the RGP library that matches only an intergenic region on human chromosome 5.** Using the BLAT tool (http://genome.ucsc.edu/cgi-bin/hgBlat), the sequence (represented in the red ellipse) was aligned to the human genome and the original scheme generated by the BLAT tool was downloaded and displayed here.

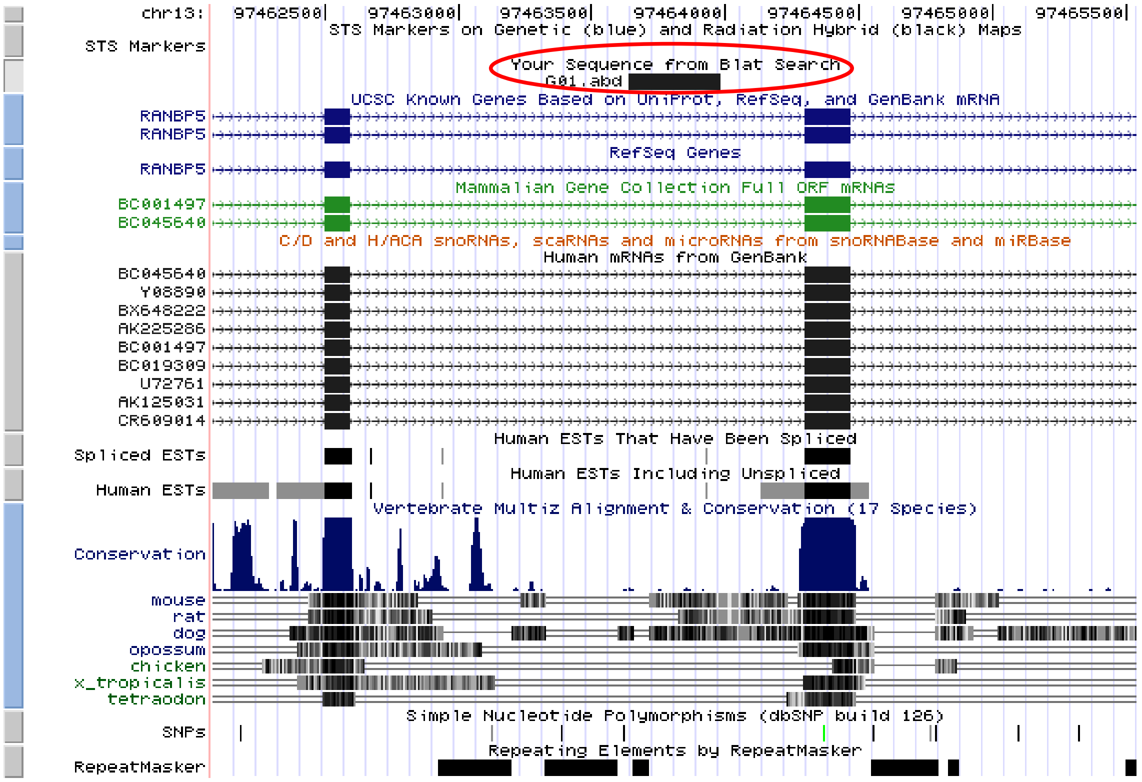


**Figure S7: Schematic view of the sequence alignment for an EST (GenBank accession number ES315919) from the RGP library that matches an intronic region (gene RANBP5) on human chromosome 13.** Using the BLAT tool (http://genome.ucsc.edu/cgi-bin/hgBlat), the sequence (represented in the red ellipse) was aligned to the human genome and the original scheme generated by the BLAT tool was downloaded and displayed here.

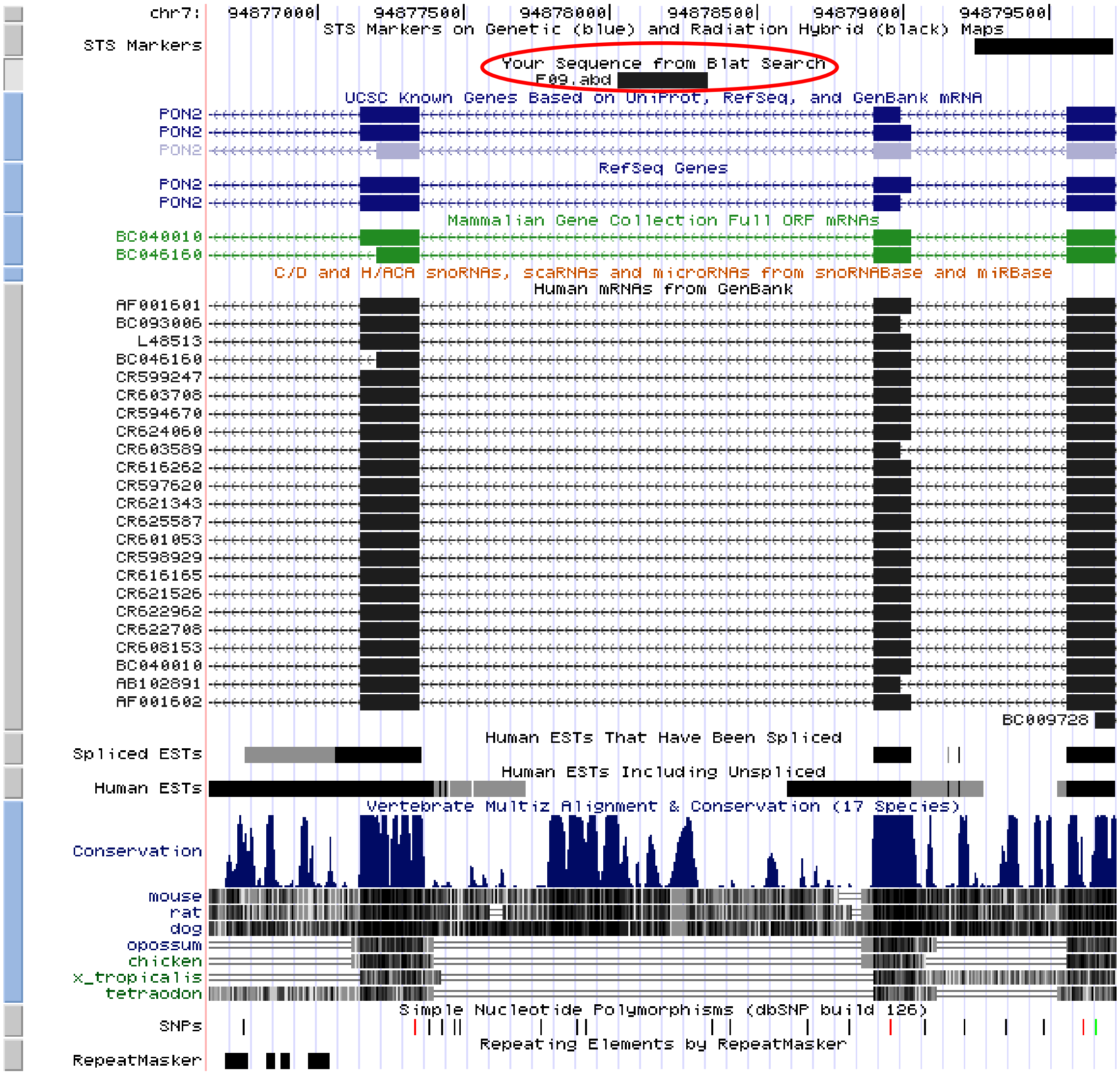


**Figure S8: Schematic view of the sequence alignment for an EST (GenBank accession number ES315722) from the RGP library that matches an intronic region (gene PON2) on human chromosome 7.** Using the BLAT tool (http://genome.ucsc.edu/cgi-bin/hgBlat), the sequence (represented in the red ellipse) was aligned to the human genome and the original scheme generated by the BLAT tool was downloaded and displayed here.

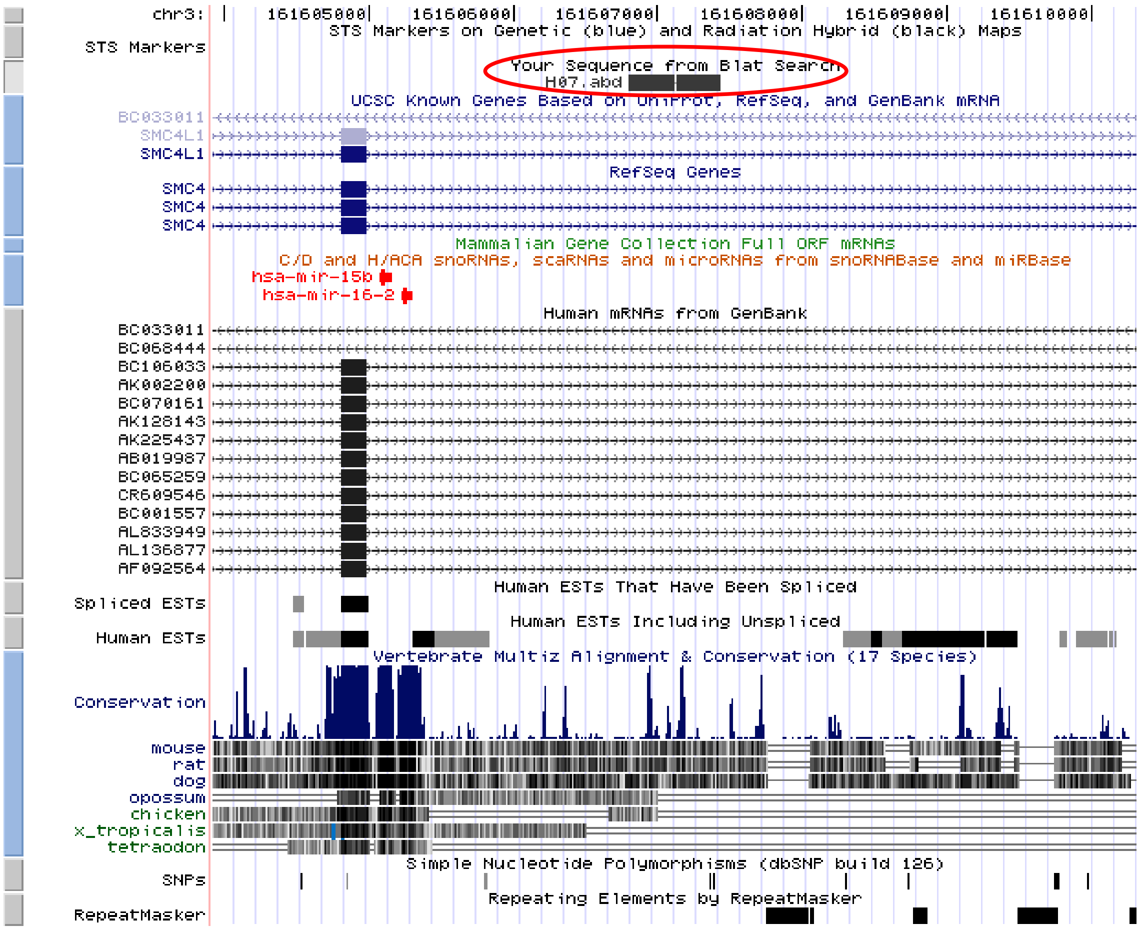


**Figure S9: Schematic view of the sequence alignment for an EST (GenBank accession number ES315872) from the RGP library that matches an intronic region (gene SMC4) on human chromosome 3.** Using the BLAT tool (http://genome.ucsc.edu/cgi-bin/hgBlat), the sequence (represented in the red ellipse) was aligned to the human genome and the original scheme generated by the BLAT tool was downloaded and displayed here.

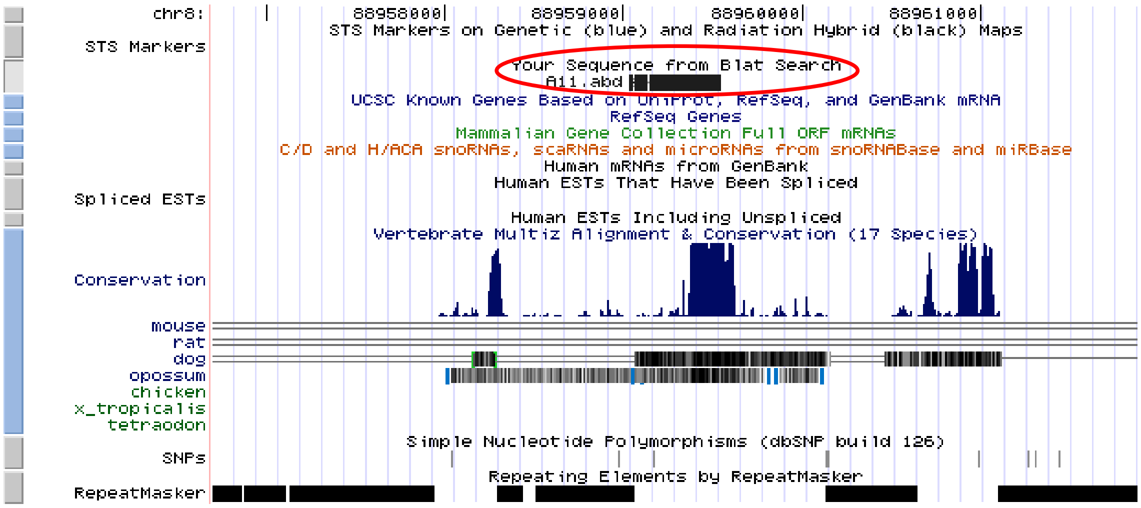


**Figure S10: Schematic view of the sequence alignment for an EST (GenBank accession number ES316146) from the Met library that matches only an intergenic region on human chromosome 8.** Using the BLAT tool ([http://genome.ucsc.edu/cgi-bin/hgBlat](http://genome.ucsc.edu/cgi-bin/hgBlat)), the sequence (represented in the red ellipse) was aligned to the human genome and the original scheme generated by the BLAT tool was downloaded and displayed here.

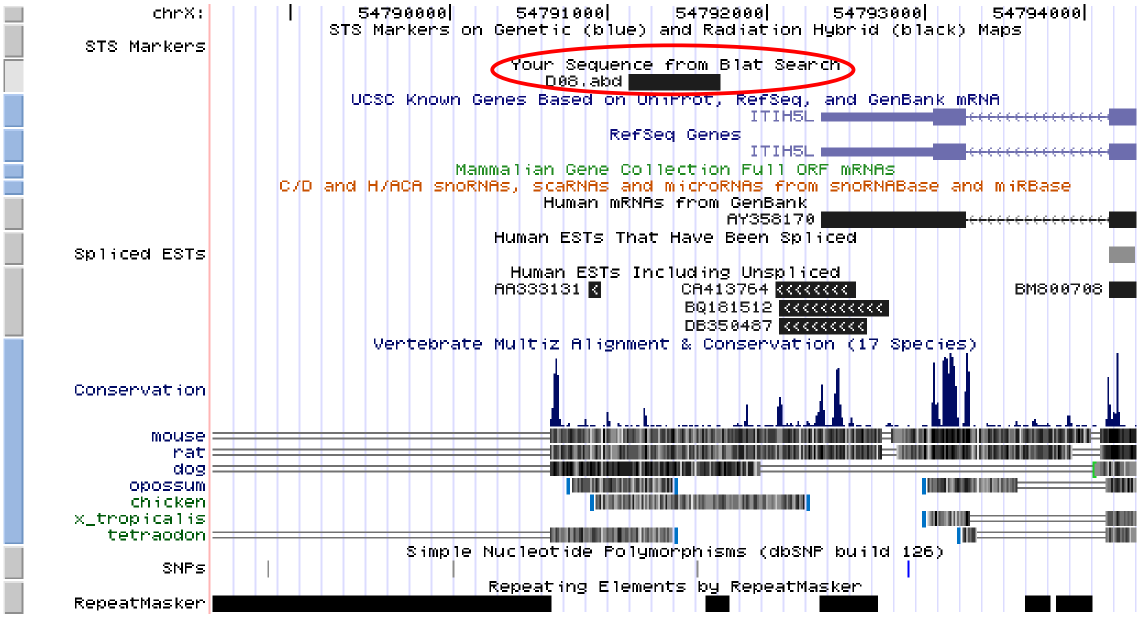


**Figure S11: Schematic view of the sequence alignment for an EST (GenBank accession number ES316173) from the Met library that matches only genomic sequence near the 3' end of the gene ITIH5L, on human chromosome X.** Using the BLAT tool ([http://genome.ucsc.edu/cgi-bin/hgBlat](http://genome.ucsc.edu/cgi-bin/hgBlat)), the sequence (represented in the red ellipse) was aligned to the human genome and the original scheme generated by the BLAT tool was downloaded and displayed here.

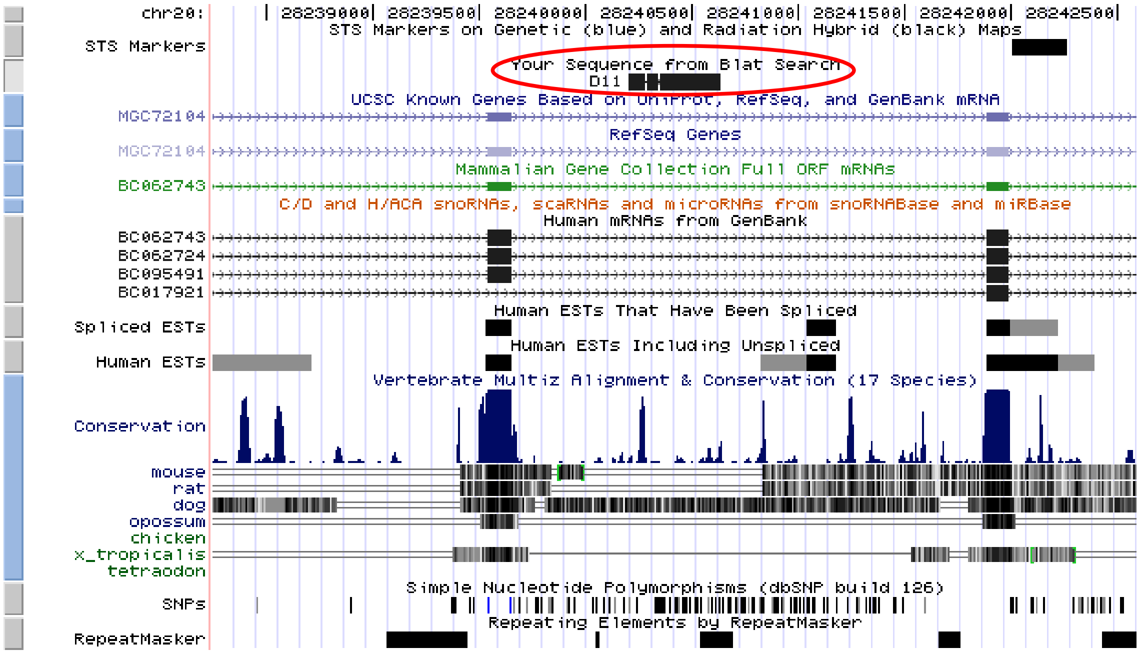


**Figure S12: Schematic view of the sequence alignment for an EST (GenBank accession number ES316377) from the Met library that matches an intronic region (gene MGC72104) on human chromosome 20.** Using the BLAT tool (http://genome.ucsc.edu/cgi-bin/hgBlat), the sequence (represented in the red ellipse) was aligned to the human genome and the original scheme generated by the BLAT tool was downloaded and displayed here

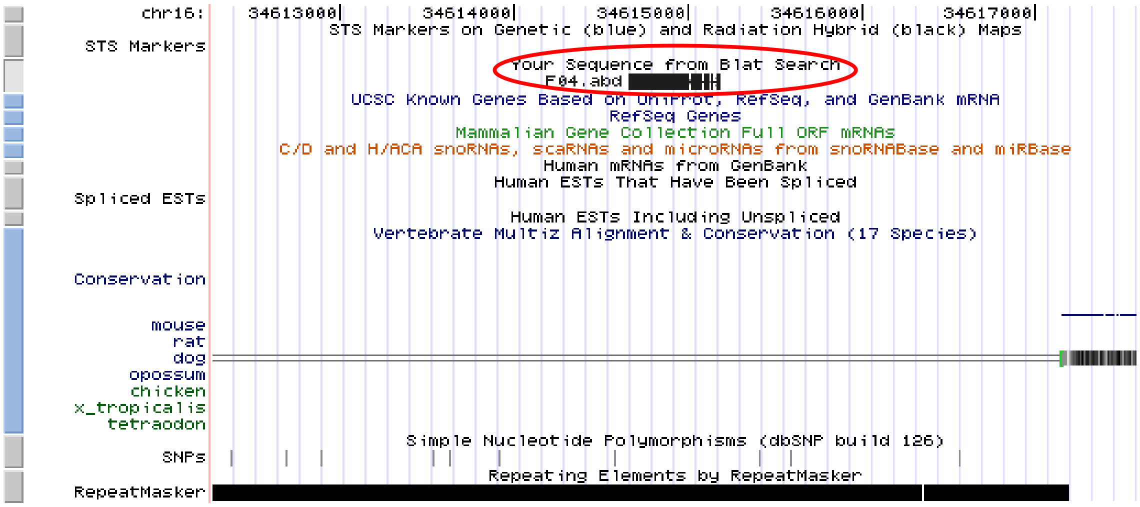


**Figure S13: Schematic view of the sequence alignment for an EST (GenBank accession number ES316257) from the Met library that matches an intergenic region on human chromosome 16.** Using the BLAT tool (http://genome.ucsc.edu/cgi-bin/hgBlat), the sequence (represented in the red ellipse) was aligned to the human genome and the original scheme generated by the BLAT tool was downloaded and displayed here.

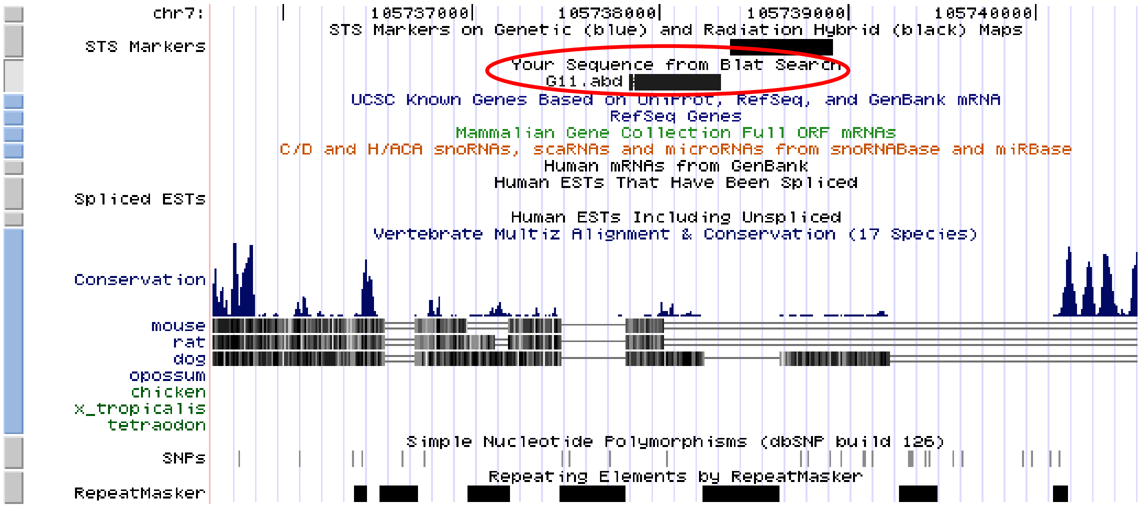


**Figure S14: Schematic view of the sequence alignment for an EST (GenBank accession number ES316330) from the Met library that matches an intergenic region on human chromosome 7.** Using the BLAT tool (http://genome.ucsc.edu/cgi-bin/hgBlat), the sequence (represented in the red ellipse) was aligned to the human genome and the original scheme generated by the BLAT tool was downloaded and displayed here.

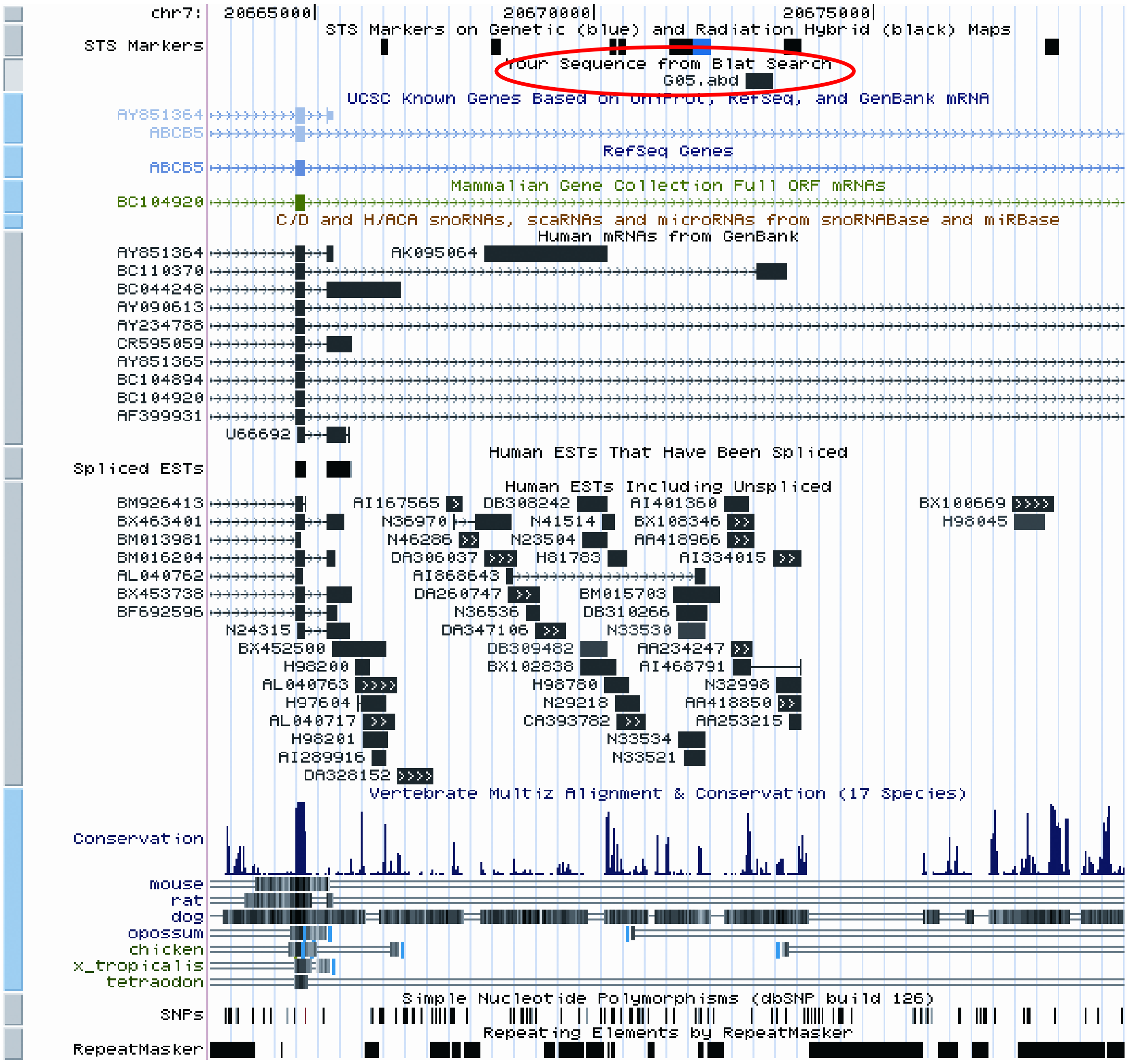


**Figure S15: Schematic view of the alignment of a sequence (GenBank accession number ES316269) from the Met library that matches and extends a putative alternative exon of the ABCB5 gene.** Using the BLAT tool (http://genome.ucsc.edu/cgi-bin/hgBlat) the sequence (represented in the red ellipse) was aligned to the human genome and the original scheme generated by the BLAT tool was downloaded and displayed here.
