# Supplementary material for: Suppression subtractive hybridization profiles of radial growth phase and metastatic melanoma cell lines reveal novel potential targets
Source: BMC Cancer. 2008 Jan 22;8:19. doi: 10.1186/1471-2407-8-19 (PMC2267200; doi:10.1186/1471-2407-8-19)
Supplement: Additional file 4 — Lists of the genes represented by redundant clones in the RGP and Met libraries. The tables contain the identifier, Gene Ontology annotation and available information about role in cancer related processes for the genes represented by redundant clones in the RGP and Met libraries. [file 1471-2407-8-19-S4.pdf]

Additional File 4: Lists of the genes represented by redundant clones in the RGP and Met libraries. The tables contain the identifier, Gene Ontology annotation and available information about role in cancer related processes for the genes represented by redundant clones in the RGP and Met libraries.

Table S3: Genes represented by a redundant number of sequences in the RGP SSH library

Symbol	Accesion number	Redundancy	GO function	Reported in cancer	Reported in melanoma
**ACAT2**	NM_005891	2	acyltransferase activity	upregulation in hepatocarcinoma [1]	ND
**ACTB**	NM_001101	3	structural constituent of cytoskeleton	actin dynamics alteration is observed in tumor progression [2]	
**ACTG1**	NM_001614	2	structural constituent of cytoskeleton	actin dynamics alteration is observed in tumor progression [2]	reduction of gamma-actin in invasive melanoma cells have been reported [3]
**ALS2CR7**	NM_139158	10	protein serine/threonine kinase activity	ND	ND
**ANLN**	NM_018685	2	actin binding	overexpressed in several tumors [4]	ND
**CFH**	NM_000186	5	innate immune response	overexpressed in ovarian carcinomas [5]	ND
**CNOT7**	NM_054026	2	transcription factor activity	ND	ND
**CXCL11**	NM_005409	2	chemokine activity	induction of CTL cells migration to tumors and apoptosis of tumor cells [6]	seems to contribute to metastasis of B16 melanoma cells [7]
**DCN**	NM_133503	8	small proteoglycan, transferase activity	tumor growth suppression [8, 9]	expressed in melanoma cell lines from different stages [10]. Inhibition of TGF-β stimulated adhesion of melanoma cells to endothelium [11]
**DDX1**	NM_004939	2	RNA helicase activity	co-amplified with MYCN and overexpressed in neuroblastoma and retinoblastoma cell lines [12]	ND
**EIF4G2**	NM_001418	2	translation initiation factor activity	downregulated in bladder carcinoma [13]	ND
**FAM33A**	AK056473	2	no evidence	ND	ND
**FAM54A**	NM_138419	2	no evidence	ND	ND
**FSTL5**	NM_020116	3	calcium ion binding	ND	ND

**GNG11**	NM_004126	2	signal transducer activity	downregulated in splenic marginal zone lymphoma [14]	ND
**HMGB2**	NM_002129	2	DNA binding, DNA bending activity, transcription factor activity	overexpressed, although associated to a better prognosis in epithelial ovarian cancer [15]	ND
**KCTD3**	NM_016121	2	voltage-gated potassium channel activity	ND	ND
**MBOAT1**	NM_175879	4	no evidence	ND	ND
**MFAP4**	NM_002404	3	calcium ion binding, protein binding, cell adhesion	ND	ND
**MYCBP**	NM_012333	2	transcription coactivator activity	upregulated in colon carcinoma cells overexpressing LEF-1 [16]	ND
**NBPF14***	NM_015383	2RGP, 1MET	no evidence	ND	ND
**NME7**	NM_013330	2	kinase activity	ND	ND
**NUCKS1**	NM_022731	2	kinase activity	ND	ND
**NUP160**	NM_015231	3	nucleocytoplasmic transporter activity	ND	ND
**OR51E2**	NM_030774	2	olfactory receptor activity	overexpressed in prostate cancer [17]	ND
**PHF20**	NM_016436	2	nucleic acid binding, trancriptional regulation	overexpressed in small cell lung cancer [18]	ND
**POGZ**	NM_015100	2	nucleic acid binding, trancriptional regulation	ND	ND
**POLD3**	D26018	2	delta DNA polymerase activity	ND	ND
**PPT1**	NM_000310	2	hydrolase activity	overexpressed in colorectal carcinoma [19]	ND
**PSMA4**	NM_002789	3	threonine endopeptidase activity	ND	ND
**RBM25**	NM_021239	2	mRNA binding, mRNA splicing	ND	ND
**RGS2**	NM_002923	2	GTPase activator activity,calmodulin binding, signal transducer activity	repressed in myeloid transformation [20]	ND
**RUNX2**	NM_001015051	2	transcription factor activity	There are evidencies showing runx proteins may act under different circumstances in both an oncogenic and tumor suppressor role [21]	ND
**SET**	NM_003011	2	histone binding, fosfatase inhibitor	histone acetyiltransferase inhibitor, up-regulated in several tumors [22]	ND

**SLC35B1**	NM_005827	2	UDP-galactose transporter activity	ND	ND
**TMTC3**	NM_181783	2RGP, 1MET	ND	ND	ND
**WDR35**	NM_001006657	2	ND	ND	ND

See references in the reference file of the supplementary material
ND: Not described

Table S4: Genes represented by a redundant number of sequences in the Met SSH library

Symbol	Accesion number	Redundancy	GO function	Reported in cancer	Reported in melanoma
**A2M**	NM_000014	12	protein carrier activity	hepatocarcinoma [23]	ND
**APOD**	NM_001647	2	lipid transporter activity	overexpressed in breast and prostate cancer [24]	the percentage of Apo-D positive tumors is higher in VGP than in RGP melanomas [25]
**C18orf19**	NM_152352	3	ND	ND	ND
**CD200**	NM_005944	3	integral to plasma membrane	suppression of tumor growth inhibition [26]	ND
**CD59**	NM_000611	9	GPI anchor binding/immune response	protects tumor cells from complement mediated lysis [27]	overexpression of CD59 in melanoma cells protects them from complement mediated lysis [28]
**CHI3L2**	NM_004000	2	hydrolase activity/carbohydrate metabolism	ND	ND
**CPM**	NM_001005502	2	GPI anchor binding, carboxypeptidase A activity, ferric iron binding , metal ion binding, metallopeptidase activity, zinc ion binding	overexpressed in tumors [29]	ND
**CTSK**	NM_000396	2	cathepsin activity/proteolysis	associated to tumor invasiviness [30]	ND
**DCT**	NM_001922	7	dopachrome isomerase activity, oxidoreductase activity/ melanin metabolism	increases proliferation of neuronal cells [31]	melanocyte/melanoma marker; increased in radioresistant melanoma cells [32]
**G3BP2**	NM_012297	3	RNA binding, receptor signaling complex scaffold activity	overexpressed in breast cancer [33]	ND
**GPM6B**	NM_001001994	3	molecular function unknown, cell differentiation	ND	ND
**HLA-DRA**	NM_019111	55	receptor activity, antigen presentation	observed as down-regulated[34] as also as up-regulated in different tumors [35]	prognostic significance of HLA-DR expresion in melanoma tumors is not clearly definied [36, 37]
**ITGB8**	NM_002214	4	receptor activity, cell-matrix adhesion	overexpressed in glioblastoma [38]	ND
**ITPR1**	NM_002222	3	ion chanel activity	ND	ND

**LAMA4**	NM_002290	2	extracellular matrix constituent, cell adhesion	angiogenesis induction [39]	ND
**LOC285628**	AL389942	3	unknown	ND	ND
**MLANA**	NM_005511	2	melanin biosynthesis (non GO)	ND	indicated as melanoma micrometastasis marker [40]
**MRPL42**	NM_172178	2	structural constituent of ribosome	ND	ND
**MTUS1**	NM_020749	3	receptor activity	considered tumor suppressor; down-regulated in pancreatic tumor [41]	ND
**NRP2**	NM_003872	2	receptor activity	expressed in gliomas and neuroblastomas [42]	expressed in melanoma [42]
**PDGFRA**	NM_006206	2	tyrosine kinase receptor	metastatic potential of oncogenic mammary epithelial cells requires an autocrine PDGF/PDGFR loop [43]	overexpressed in melanoma cells [44]
**PLP1**	NM_000533	25	structural molecule activity; involved in myelination	overexpressed in leiomyomas [45]	ND
**POMP**	NM_015932	2	protein folding, immune response	interacting partners of the human papillomavirus 16 transcription/replication factor E2 [46]	ND
**RDX**	NM_002906	2	actin binding, cytoskeletal anchoring	up-regulated in renal cell carcinoma [47]	ND
**SERPINE2**	NM_006216	4	serine-type endopeptidase inhibitor activity	overexpressed in pancreatic tumor[48]	ND
**SGK**	NM_005627	2	protein serine/threonine kinase activity	overexpressed in extraskeletal myxoid chondrosarcomas [49]	ND
**SLC5A4**	NM_014227	2	symporter activity	ND	ND
**SPP1**	NM_000582	2	cytokine activity, growth factor activity	involved in tumor progression and metastasis [50]	overexpressed in PTEN mutant melanomas [51]
**TDO2**	NM_005651	8	tryptophan 2,3-dioxygenase activity	overexpressed in esophageal adenocarcinoma [52]	ND
**TM4SF1**	NM_014220	10	molecular function unknown, integral to membrane	may be involved in cancer invasion and metastasis [53]	ND
**TYR**	NM_000372	2	monooxygenase activity; melanin biosynthesis	ND	used as molecular marker for detection circulating melanoma cells [54]

**TYRP1**	NM_000550	2	monooxygenase activity; melanin biosynthesis	ND	decreases the tyrosinase mediated cell death [55]

See references in the reference file of the supplementary material
ND: Not described

References

1. Song BL, Wang CH, Yao XM, Yang L, Zhang WJ, Wang ZZ, Zhao XN, Yang JB, Qi W, Yang XY, et al: **Human acyl-CoA:cholesterol acyltransferase 2 gene expression in intestinal Caco-2 cells and in hepatocellular carcinoma.** *Biochem J* 2006, **394:**617-626.

2. Lambrechts A, Van Troys M, Ampe C: **The actin cytoskeleton in normal and pathological cell motility.** *Int J Biochem Cell Biol* 2004, **36:**1890-1909.

3. Gutgemann A, Golob M, Muller S, Buettner R, Bosserhoff AK: **Isolation of invasion-associated cDNAs in melanoma.** *Arch Dermatol Res* 2001, **293:**283-290.

4. Hall PA, Todd CB, Hyland PL, McDade SS, Grabsch H, Dattani M, Hillan KJ, Russell SE: **The septin-binding protein anillin is overexpressed in diverse human tumors.** *Clin Cancer Res* 2005, **11:**6780-6786.

5. Urzua U, Roby KF, Gangi LM, Cherry JM, Powell JI, Munroe DJ: **Transcriptomic analysis of an in vitro murine model of ovarian carcinoma: functional similarity to the human disease and identification of prospective tumoral markers and targets.** *J Cell Physiol* 2006, **206:**594-602.

6. Berencsi K, Meropol NJ, Hoffman JP, Sigurdson E, Giles L, Rani P, Somasundaram R, Zhang T, Kalabis J, Caputo L, et al: **Colon carcinoma cells induce CXCL11-dependent migration of CXCR3-expressing cytotoxic T lymphocytes in organotypic culture.** *Cancer Immunol Immunother* 2006.

7. Kawada K, Sonoshita M, Sakashita H, Takabayashi A, Yamaoka Y, Manabe T, Inaba K, Minato N, Oshima M, Taketo MM: **Pivotal role of CXCR3 in melanoma cell metastasis to lymph nodes.** *Cancer Res* 2004, **64:**4010-4017.

8. Seidler DG, Goldoni S, Agnew C, Cardi C, Thakur ML, Owens RT, McQuillan DJ, Iozzo RV: **Decorin protein core inhibits in vivo cancer growth and metabolism by hindering epidermal growth factor receptor function and triggering apoptosis via caspase-3 activation.** *J Biol Chem* 2006, **281:**26408-26418.

9. Stander M, Naumann U, Wick W, Weller M: **Transforming growth factor-beta and p-21: multiple molecular targets of decorin-mediated suppression of neoplastic growth.** *Cell Tissue Res* 1999, **296:**221-227.

10. Ladanyi A, Gallai M, Paku S, Nagy JO, Dudas J, Timar J, Kovalszky I: **Expression of a decorin-like moleculein human melanoma.** *Pathol Oncol Res* 2001, **7:**260-266.

11. Teti A, De Giorgi A, Spinella MT, Migliaccio S, Canipari R, Onetti Muda A, Faraggiana T: **Transforming growth factor-beta enhances adhesion of melanoma cells to the endothelium in vitro.** *Int J Cancer* 1997, **72:**1013-1020.

12. Godbout R, Packer M, Bie W: **Overexpression of a DEAD box protein (DDX1) in neuroblastoma and retinoblastoma cell lines.** *J Biol Chem* 1998, **273:**21161-21168.

13. Buim ME, Soares FA, Sarkis AS, Nagai MA: **The transcripts of SFRP1,CEP63 and EIF4G2 genes are frequently downregulated in transitional cell carcinomas of the bladder.** *Oncology* 2005, **69:**445-454.

14. Ruiz-Ballesteros E, Mollejo M, Rodriguez A, Camacho FI, Algara P, Martinez N, Pollan M, Sanchez-Aguilera A, Menarguez J, Campo E, et al: **Splenic marginal zone lymphoma: proposal of new diagnostic and prognostic markers identified after tissue and cDNA microarray analysis.** *Blood* 2005, **106:**1831-1838.

15. Ouellet V, Le Page C, Guyot MC, Lussier C, Tonin PN, Provencher DM, Mes-Masson AM: **SET complex in serous epithelial ovarian cancer.** *Int J Cancer* 2006, **119:**2119-2126.

16. Jung HC, Kim K: **Identification of MYCBP as a beta-catenin/LEF-1 target using DNA microarray analysis.** *Life Sci* 2005, **77:**1249-1262.

17. Xu LL, Sun C, Petrovics G, Makarem M, Furusato B, Zhang W, Sesterhenn IA, McLeod DG, Sun L, Moul JW, Srivastava S: **Quantitative expression profile of PSGR in prostate cancer.** *Prostate Cancer Prostatic Dis* 2006, **9:**56-61.

18. Taniwaki M, Daigo Y, Ishikawa N, Takano A, Tsunoda T, Yasui W, Inai K, Kohno N, Nakamura Y: **Gene expression profiles of small-cell lung cancers: molecular signatures of lung cancer.** *Int J Oncol* 2006, **29:**567-575.

19. Tsukamoto T, Iida J, Dobashi Y, Furukawa T, Konishi F: **Overexpression in colorectal carcinoma of two lysosomal enzymes, CLN2 and CLN1, involved in neuronal ceroid lipofuscinosis.** *Cancer* 2006, **106:**1489-1497.

20. Schwable J, Choudhary C, Thiede C, Tickenbrock L, Sargin B, Steur C, Rehage M, Rudat A, Brandts C, Berdel WE, et al: **RGS2 is an important target gene of Flt3-ITD mutations in AML and functions in myeloid differentiation and leukemic transformation.** *Blood* 2005, **105:**2107-2114.

21. Thomas D, Kansara M: **Epigenetic modifications in osteogenic differentiation and transformation.** *J Cell Biochem* 2006, **98:**757-769.

22. Cervoni N, Detich N, Seo SB, Chakravarti D, Szyf M: **The oncoprotein Set/TAF-1beta, an inhibitor of histone acetyltransferase, inhibits active demethylation of DNA, integrating DNA methylation and transcriptional silencing.** *J Biol Chem* 2002, **277:**25026-25031.

23. Kurokawa Y, Matoba R, Takemasa I, Nakamori S, Tsujie M, Nagano H, Dono K, Umeshita K, Sakon M, Ueno N, et al: **Molecular features of non-B, non-C hepatocellular carcinoma: a PCR-array gene expression profiling study.** *J Hepatol* 2003, **39:**1004-1012.

24. Xu S, Venge P: **Lipocalins as biochemical markers of disease.** *Biochim Biophys Acta* 2000, **1482:**298-307.

25. Miranda E, Vizoso F, Martin A, Quintela I, Corte MD, Segui ME, Ordiz I, Merino AM: **Apolipoprotein D expression in cutaneous malignant melanoma.** *J Surg Oncol* 2003, **83:**99-105.

26. Gorczynski RM, Chen Z, Hu J, Kai Y, Lei J: **Evidence of a role for CD200 in regulation of immune rejection of leukaemic tumour cells in C57BL/6 mice.** *Clin Exp Immunol* 2001, **126:**220-229.

27. Fishelson Z, Donin N, Zell S, Schultz S, Kirschfink M: **Obstacles to cancer immunotherapy: expression of membrane complement regulatory proteins (mCRPs) in tumors.** *Mol Immunol* 2003, **40:**109-123.

28. Coral S, Fonsatti E, Sigalotti L, De Nardo C, Visintin A, Nardi G, Colizzi F, Colombo MP, Romano G, Altomonte M, Maio M: **Overexpression of protectin (CD59) down-modulates the susceptibility of human melanoma cells to homologous complement.** *J Cell Physiol* 2000, **185:**317-323.

29. Pessoa LG, da Silva ID, Baptista HA, Pesquero JL, Paiva AC, Bader M, Pesquero JB: **Molecular structure and alternative splicing of the human carboxypeptidase M gene.** *Biol Chem* 2002, **383:**263-269.

30. Rapa I, Volante M, Cappia S, Rosas R, Scagliotti GV, Papotti M: **Cathepsin K is selectively expressed in the stroma of lung adenocarcinoma but not in bronchioloalveolar carcinoma. A useful marker of invasive growth.** *Am J Clin Pathol* 2006, **125:**847-854.

31. Jiao Z, Zhang ZG, Hornyak TJ, Hozeska A, Zhang RL, Wang Y, Wang L, Roberts C, Strickland FM, Chopp M: **Dopachrome tautomerase (Dct) regulates neural progenitor cell proliferation.** *Dev Biol* 2006, **296:**396-408.

32. Pak BJ, Lee J, Thai BL, Fuchs SY, Shaked Y, Ronai Z, Kerbel RS, Ben-David Y: **Radiation resistance of human melanoma analysed by retroviral insertional mutagenesis reveals a possible role for dopachrome tautomerase.** *Oncogene* 2004, **23:**30-38.

33. French J, Stirling R, Walsh M, Kennedy HD: **The expression of Ras-GTPase activating protein SH3 domain-binding proteins, G3BPs, in human breast cancers.** *Histochem J* 2002, **34:**223-231.

34. Matoba K, Iizuka N, Gondo T, Ishihara T, Yamada-Okabe H, Tamesa T, Takemoto N, Hashimoto K, Sakamoto K, Miyamoto T, et al: **Tumor HLA-DR expression linked to early intrahepatic recurrence of hepatocellular carcinoma.** *Int J Cancer* 2005, **115:**231-240.

35. Rangel LB, Agarwal R, Sherman-Baust CA, Mello-Coelho V, Pizer ES, Ji H, Taub DD, Morin PJ: **Anomalous expression of the HLA-DR alpha and beta chains in ovarian and other cancers.** *Cancer Biol Ther* 2004, **3:**1021-1027.

36. Kalogeraki A, Garbagnati F, Darivianaki K, Delides GS, Santinami M, Stathopoulos EN, Zoras O: **HSP-70, C-myc and HLA-DR expression in patients with cutaneous malignant melanoma metastatic in lymph nodes.** *Anticancer Res* 2006, **26:**3551-3554.

37. Ricaniadis N, Kataki A, Agnantis N, Androulakis G, Karakousis CP: **Long-term prognostic significance of HSP-70, c-myc and HLA-DR expression in patients with malignant melanoma.** *Eur J Surg Oncol* 2001, **27:**88-93.

38. Riemenschneider MJ, Mueller W, Betensky RA, Mohapatra G, Louis DN: **In situ analysis of integrin and growth factor receptor signaling pathways in human glioblastomas suggests overlapping relationships with focal adhesion kinase activation.** *Am J Pathol* 2005, **167:**1379-1387.

39. Lian J, Dai X, Li X, He F: **Identification of an active site on the laminin alpha4 chain globular domain that binds to alphavbeta3 integrin and promotes angiogenesis.** *Biochem Biophys Res Commun* 2006, **347:**248-253.

40. Lewis TB, Robison JE, Bastien R, Milash B, Boucher K, Samlowski WE, Leachman SA, Dirk Noyes R, Wittwer CT, Perreard L, Bernard PS: **Molecular classification of melanoma using real-time quantitative reverse transcriptase-polymerase chain reaction.** *Cancer* 2005, **104:**1678-1686.

41. Seibold S, Rudroff C, Weber M, Galle J, Wanner C, Marx M: **Identification of a new tumor suppressor gene located at chromosome 8p21.3-22.** *FASEB J* 2003, **17:**1180-1182.

42. Bielenberg DR, Pettaway CA, Takashima S, Klagsbrun M: **Neuropilins in neoplasms: expression, regulation, and function.** *Exp Cell Res* 2006, **312:**584-593.

43. Jechlinger M, Sommer A, Moriggl R, Seither P, Kraut N, Capodiecci P, Donovan M, Cordon-Cardo C, Beug H, Grunert S: **Autocrine PDGFR signaling promotes mammary cancer metastasis.** *J Clin Invest* 2006, **116:**1561-1570.

44. McGary EC, Onn A, Mills L, Heimberger A, Eton O, Thomas GW, Shtivelband M, Bar-Eli M: **Imatinib mesylate inhibits platelet-derived growth factor receptor phosphorylation of melanoma cells but does not affect tumorigenicity in vivo.** *J Invest Dermatol* 2004, **122:**400-405.

45. Skubitz KM, Skubitz AP: **Differential gene expression in uterine leiomyoma.** *J Lab Clin Med* 2003, **141:**297-308.

46. Boner W, Morgan IM: **Novel cellular interacting partners of the human papillomavirus 16 transcription/replication factor E2.** *Virus Res* 2002, **90:**113-118.

47. Craven RA, Stanley AJ, Hanrahan S, Dods J, Unwin R, Totty N, Harnden P, Eardley I, Selby PJ, Banks RE: **Proteomic analysis of primary cell lines**

identifies protein changes present in renal cell carcinoma. *Proteomics* 2006, **6:**2853-2864.

48. Buchholz M, Biebl A, Neesse A, Wagner M, Iwamura T, Leder G, Adler G, Gress TM: **SERPINE2 (protease nexin I) promotes extracellular matrix production and local invasion of pancreatic tumors in vivo.** *Cancer Res* 2003, **63:**4945-4951.

49. Poulin H, Filion C, Ladanyi M, Labelle Y: **Serum- and glucocorticoid-regulated kinase 1 (SGK1) induction by the EWS/NOR1(NR4A3) fusion protein.** *Biochem Biophys Res Commun* 2006, **346:**306-313.

50. Rangaswami H, Bulbule A, Kundu GC: **Osteopontin: role in cell signaling and cancer progression.** *Trends Cell Biol* 2006, **16:**79-87.

51. Packer L, Pavey S, Parker A, Stark M, Johansson P, Clarke B, Pollock P, Ringner M, Hayward N: **Osteopontin is a downstream effector of the PI3-kinase pathway in melanomas that is inversely correlated with functional PTEN.** *Carcinogenesis* 2006, **27:**1778-1786.

52. Cheng P, Gong J, Wang T, Jie C, Liu GS, Zhang R: **Gene expression in Barrett's esophagus and reflux esophagitis induced by gastroduodenoesophageal reflux in rats.** *World J Gastroenterol* 2005, **11:**3277-3280.

53. Kao YR, Shih JY, Wen WC, Ko YP, Chen BM, Chan YL, Chu YW, Yang PC, Wu CW, Roffler SR: **Tumor-associated antigen L6 and the invasion of human lung cancer cells.** *Clin Cancer Res* 2003, **9:**2807-2816.

54. Prichard RS, Dijkstra B, McDermott EW, Hill AD, O'Higgins NJ: **The role of molecular staging in malignant melanoma.** *Eur J Surg Oncol* 2003, **29:**306-314.

55. Rad HH, Yamashita T, Jin HY, Hirosaki K, Wakamatsu K, Ito S, Jimbow K: **Tyrosinase-related proteins suppress tyrosinase-mediated cell death of melanocytes and melanoma cells.** *Exp Cell Res* 2004, **298:**317-328.
