# Supplementary material for: Suppression subtractive hybridization profiles of radial growth phase and metastatic melanoma cell lines reveal novel potential targets
Source: BMC Cancer. 2008 Jan 22;8:19. doi: 10.1186/1471-2407-8-19 (PMC2267200; doi:10.1186/1471-2407-8-19)
Supplement: Additional file 5 — Search for the expression profile of the genes identified in the RGP and Met libraries in a melanoma microarray study performed by Hoek et al. [92]. Lists of the genes represented in the RGP and Met libraries that were detected as differentially expressed between melanocytes and melanoma cell lines in the above cited study. [file 1471-2407-8-19-S5.pdf]

Additional File 5: Search for the expression profile of the genes identified in the RGP and Met libraries in a melanoma microarray study performed by Hoek et al. [92]. The tables contain the lists of the genes represented in the RGP and Met libraries that were detected as differentially expressed between melanocytes and melanoma cell lines in the above cited study.

Table S5: RGP library genes detected as up-regulated in melanoma cells in comparison to melanocytes in the study by Hoek et al [92]*.

Gene name	Symbol	Accession number
amphoterin induced gene 2	**AMIGO2**	NM_181847
angiopoietin 1	**ANGPT1**	NM_001146
adenylate cyclase-associated protein, 2	**CAP2**	AL832154
decorin	**DCN**	NM_133503
ectonucleotide pyrophosphatase/phosphodiesterase 1	**ENPP1**	NM_006208
histone deacetylase 1	**HDAC1**	NM_004964
lumican	**LUM**	NM_002345
platelet derived growth factor C	**PDGFC**	NM_016205
paternally expressed 10	**PEG10**	NM_015068
pogo transposable element with ZNF domain	**POGZ**	NM_015100
prostaglandin-endoperoxide synthase 2	**PTGS2**	NM_000963
secretogranin II (chromogranin C)	**SCG2**	NM_003469
solute carrier family 38, member 2	**SLC38A2**	NM_018976

Table S6: Met library genes detected as differentially expressed between melanocytes and melanoma cells in the study by Hoek et al [92]*.

	Gene name	Symbol	Accession number
**Up-regulated in melanoma cells**	aldehyde dehydrogenase 1 family, member A3	**ALDH1A3**	NM_000693
	apolipoprotein D	**APOD**	NM_001647
	calumenin	**CALU**	NM_001219
	connective tissue growth factor	**CTGF**	U14750
	EphA3	**EPHA3**	NM_005233
	fibronectin 1	**FN1**	NM_002026
	Homo sapiens G protein-coupled receptor 126	**GPR126**	NM_020455
	major histocompatibility complex, class II	**HLA-DRA**	NM_019111
	interleukin 1 receptor accessory protein	**IL1RAP**	NM_002182
	integrin, alpha 6	**ITGA6**	NM_000210
	inositol 1,4,5-triphosphate receptor, type 1	**ITPR1**	NM_002222
	laminin, alpha 4	**LAMA4**	NM_002290
	lysosomal-associated membrane protein 2	**LAMP2**	NM_002294
	neuronal cell adhesion molecule	**NRCAM**	NM_005010
	prion protein (p27-30)	**PRNP**	NM_183079
	solute carrier family 38, member 2	**SLC38A2**	NM_018976
	transforming growth factor, beta-induced, 68kDa	**TGFBI**	NM_000358
	tissue inhibitor of metalloproteinase 3	**TIMP3**	NM_000362
	transmembrane 4 superfamily member 1	**TM4SF1**	NM_014220
**Down-regulated in melanoma cells**	eukaryotic translation initiation factor 1A, Y-linked	**EIF1AY**	NM_004681
	interferon-induced protein with tetratricopeptide repeats 1	**IFIT1**	NM_001548
	solute carrier family 5 (low affinity glucose cotransporter), member 4	**SLC5A4**	NM_014227
	tyrosinase (oculocutaneous albinism IA)	**TYR**	NM_000372

* The reference number corresponds to the number of the reference list of the main text.
