# Supplementary material for: Suppression subtractive hybridization profiles of radial growth phase and metastatic melanoma cell lines reveal novel potential targets
Source: BMC Cancer. 2008 Jan 22;8:19. doi: 10.1186/1471-2407-8-19 (PMC2267200; doi:10.1186/1471-2407-8-19)
Supplement: Additional file 6 — Search for the expression profile of the genes identified in the RGP and Met libraries in the proteomics and SAGE analyses performed by de Souza et al. [93]. Lists of the genes represented in the RGP and Met libraries that were detected as differentially expressed between non-tumorigenic and tumorigenic murine melanocytic cell lines in the above cited study. [file 1471-2407-8-19-S6.pdf]

Additional File 6: Search for the expression profile of the genes identified in the RGP and Met libraries in the proteomics and SAGE analyses performed by de Souza et al. [93]. The table lists the genes represented in the RGP and Met libraries that were detected as differentially expressed between non-tumorigenic and tumorigenic murine melanocytic cell lines in the above cited study.

Table S7: Genes from the SSH libraries also detected as differentialy expressed between non-tumorigenic and tumorigenic murine melanocytic cell lines in the proteomics and SAGE analyses performed by de Souza et al. [93]*

		RGP library genes	Met Library genes
SAGE	up-regulated in the nontumoral melanocytic cell	ATF4	CALU
			EIF1AY
			TYRP1
	up-regulated in the melanoma cell line TM1	RPS27A	HSPA5
		LSM5	NPM1
		ACTG1	DDX5
Proteomics	up-regulated in the melanoma cell lines TM1 and TM5	ACTB	NPM1

* The reference number corresponds to the number of the reference list of the main text.
